# The *GAMYB-like* gene *SlMYB33* mediates flowering and pollen development in tomato

**DOI:** 10.1038/s41438-020-00366-1

**Published:** 2020-09-01

**Authors:** Yan Zhang, Bo Zhang, Tongwen Yang, Jie Zhang, Bin Liu, Xiangqiang Zhan, Yan Liang

**Affiliations:** 1 College of Horticulture, Northwest A&F University, Yangling, 712100 Shaanxi P. R. China; 2 School of Agriculture and Biology, Shanghai Jiao Tong University, Shanghai, 200240 China

**Keywords:** Plant development, Developmental biology

## Abstract

GAMYBs are positive GA signaling factors that exhibit essential functions in reproductive development, particularly in anther and pollen development. However, there is no direct evidence of the regulation of any *GAMYB* in these biological processes in tomato (*Solanum lycopersicum*). Here, we identified a tomato *GAMYB-like* gene, *SlMYB33*, and characterized its specific roles. *SlMYB33* is predominately expressed in the stamens and pistils. During flower development, high mRNA abundance of *SlMYB33* is detected in both male and female organs, such as microspore mother cells, anthers, pollen grains, and ovules. Silencing of *SlMYB33* leads to delayed flowering, aberrant pollen viability, and poor fertility in tomato. Histological analyses indicate that *SlMYB33* exerts its function in pollen development in the mature stage. Further transcriptomic analyses imply that the knockdown of *SlMYB33* significantly inhibits the expression of genes related to flowering in shoot apices, and alters the transcription of genes controlling sugar metabolism in anthers. Taken together, our study suggests that *SlMYB33* regulates tomato flowering and pollen maturity, probably by modulating the expression of genes responsible for flowering and sugar metabolism, respectively.

## Introduction

*GAMYB* encodes an R2R3-MYB transcription factor that acts as a positive gibberellin (GA) signaling factor^1^. The first *GAMYB* was isolated from aleurone cells in barley (*Hordeum vulgare*) and controls the expression of most GA-inducible genes^2–4^. *GAMYB* has been shown to be a target of microRNA 159 (miR159), a highly conserved miRNA family in the common ancestor of all embryophytes^5^. MiR159 controls the cleavage of *GAMYB* mRNA^6–8^.

A series of studies have reported the differing roles of *GAMYBs* in the regulation of flowering. *Lolium temulentum LtGAMYB* is mainly expressed in the shoot tip, and its transcript level is upregulated in parallel with an increased GA content during the floral transition^9^. The *GAMYB* family has three members in *Arabidopsis*: *AtMYB33*, *AtMYB65*, and *AtMYB101*^10^. When *Arabidopsis* plants are transferred from short-day to long-day conditions or treated with exogenous GA under short days, flowering occurs, and the expression of *AtMYB33* and *LEAFY* (*LFY*), the floral meristem-identity gene, increases in the shoot apex at that time. Furthermore, the expression timing and pattern of *AtMYB33* precede and overlap with those of *LFY* at the shoot apex, and *AtMYB33* can bind to a specific 8-bp sequence in the *LFY* promoter^10^. These observations imply that *GAMYB* may be involved in GA-regulated flowering via the transcriptional activation of the *LFY* gene. However, several studies have suggested that loss-of-function mutants of *GAMYBs* do not exhibit altered flowering in *Arabidopsis* and rice (*Oryza sativa*)^11,12^. Moreover, the manipulation of miR159 controls flowering time in some cases by regulating the expression level of its target *GAMYB*. For instance, the overexpression of miR159 delays flowering by inhibiting *AtMYB33* under a short-day photoperiod in *Arabidopsis*^6^, but another line of evidence showed that the flowering time remains normal under long-day conditions in miR159-overexpressing plants, which causes the downregulation of *AtMYB101*^8^. In contrast, the *Arabidopsis mir159ab* double mutant displays late flowering under short days, but shows no alteration of flowering time under long days^11^. In addition, the repression of miR159, which increases *GAMYB* expression, accelerates flowering in gloxinia (*Sinningia speciosa*)^13^ but delays flowering in tobacco (*Nicotiana tabacum*)^14^.

*GAMYBs* also play an essential role in flower development, particularly in stamen development. In barley, the expression of *HvGAMYB* is predominately detected in the anthers, and *HvGAMYB* overexpression leads to reduced anther length, failure of anther dehiscence, and male sterility, mimicking the effect of excessive GA on flower development^15^. In rice, *OsGAMYB* is strongly expressed in the shoot apex, stamen primordia, and tapetum cells at the reproductive stage. Loss of function of *OsGAMYB* results in some defects in anther and pollen development^12,16^. Moreover, *OsGAMYB* has been found to participate in the GA-regulated formation of exines and Ubisch bodies and the programmed cell death (PCD) of tapetal cells; specifically, the direct induction of *CYP703A3* by *OsGAMYB* is key to the formation of exines and Ubisch bodies^17^. The *Arabidopsis myb33 myb65* double mutant shows shorter stamens that fail to fully extend to the stigma, and displays defective pollen development owing to the hypertrophy of the tapetum at the microspore mother cell (MMC) stage, resulting in male sterility. However, single mutants (*myb33* or *myb65*) do not exhibit aberrant phenotypes, indicating crucial functional redundancy between *AtMYB33* and *AtMYB65*^7^. The overexpression of miR159, repressing its target *AtMYB33*, also causes anther defects and male sterility^6^. There are three *GAMYB*-like genes in cucumber (*Cucumis sativus*): *CsGAMYB1*, *CsGAMYB2*, and *CsGAMYB3*. The silencing of *CsGAMYB1* decreases the number of male flower nodes and increases that of female flower nodes, leading to a significant change in cucumber sex expression^18,19^. In tomato, the *GAMYB* homolog *SlGAMYB1* has been identified, which is expressed in the embryo and endosperm during seed germination and in young vegetative tissues^20^.

Therefore, although *GAMYBs* have been studied extensively in plants, there is no direct evidence of the regulation of any *GAMYB* (including *SlGAMYB1*) during flowering and pollen development in tomato, which is an important commercial crop and a model species for studying flower and fruit development^21,22^. Therefore, in the present study, we revealed the specific roles of *SlMYB33*, a *GAMYB-like* gene, in flowering and pollen development in tomato. We found that *SlMYB33* was mainly expressed in stamens and pistils. The knockdown of *SlMYB33* resulted in delayed flowering, aberrant pollen maturity, and remarkably decreased fertility. RNA-Seq analyses revealed that *SlMYB33* probably achieves its positive roles in tomato flowering and pollen maturity by regulating the expression of genes involved in flowering and sugar metabolism, respectively.

## Results

### Identification of the *GAMYB-like* gene *SlMYB33* from tomato

**Fig. 1:**
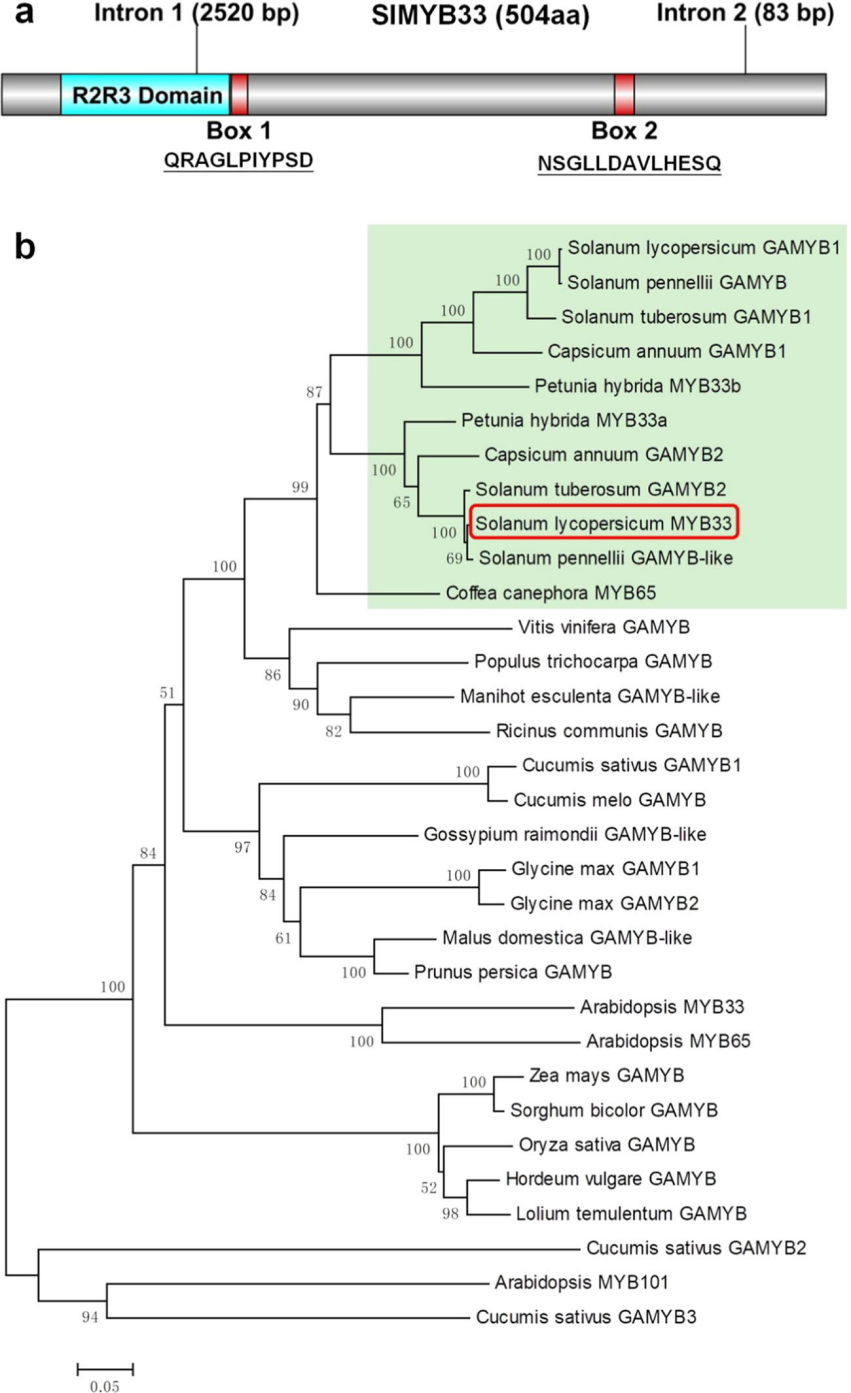
Structural and phylogenetic analyses of SlMYB33. **a** Protein domain structure of SlMYB33. **b** Phylogenetic analysis of GAMYB homologs in different species. SlMYB33 is indicated in the red box. The species in the light-green box all belong to the Solanaceae family. The gene ID of each GAMYB protein used for this analysis is provided in the “Accession Numbers” list

Following a BLAST search in the Solanaceae Genomics Network, we identified two *GAMYB-like* genes, *SlGAMYB1* (*Solyc01g009070*) and *SlMYB33* (*Solyc06g073640*), in tomato. Since the *SlGAMYB1* gene has been reported previously^20^, we focused on *SlMYB33* in this study. *SlMYB33* contains three exons and two introns (Fig. 1a), similar to the *GAMYB* orthologs found in barley, *Arabidopsis*, and cucumber^10,18^. The full-length CDS of *SlMYB33* consists of 1515 bp and encodes 504 amino acids. Previous studies have reported that GAMYBs belong to the R2R3-MYB family and contain helix-turn-helix repeats R2 and R3 and three typically conserved motifs, Box 1, Box 2, and Box 3^10,23^. Analyses of the protein domain structure and the sequence alignments of the amino acid residues revealed that SlMYB33 also includes an R2R3-repeat DNA-binding domain and conserved Box 1 (QRAGLPIYPSD) and Box 2 (NSGLLDAVLHESQ) motifs, but no Box 3 (Fig. 1a; Supplementary Fig. S1). SlMYB33 shares the highest overall identity with cucumber CsGAMYB1 (40.49%); however, within the R2R3 sequence alone, the identity of AtMYB33, AtMYB65, CsGAMYB1, and HvGAMYB with SlMYB33 is 84.62%, 83.65%, 90.38%, and 89.42%, respectively (Supplementary Fig. S1).

To reveal the evolutionary relationships between SlMYB33 and other GAMYB homologs, 32 predicted GAMYB proteins from 22 species were included in a phylogenetic analysis. As shown in Fig. 1b, the GAMYBs of Solanaceae species, including tomato, potato (*Solanum tuberosum*), pepper (*Capsicum annuum*), *Petunia hybrida*, and *Coffea canephora*, were placed in a single group, suggesting that they may share a common origin. Among these sequences, SlMYB33, *Solanum pennellii* (a wild tomato species) GAMYB-like, and potato GAMYB2 fall into the same clade, which is highly distinct from that of *Arabidopsis* MYB33 and MYB65. In addition, SlGAMYB1 is located in the same cluster with *Solanum pennellii* GAMYB and potato GAMYB1. These results implied that tomato *SlMYB33* belongs to the *GAMYB* family.

### *SlMYB33* expression pattern in tomato

**Fig. 2:**
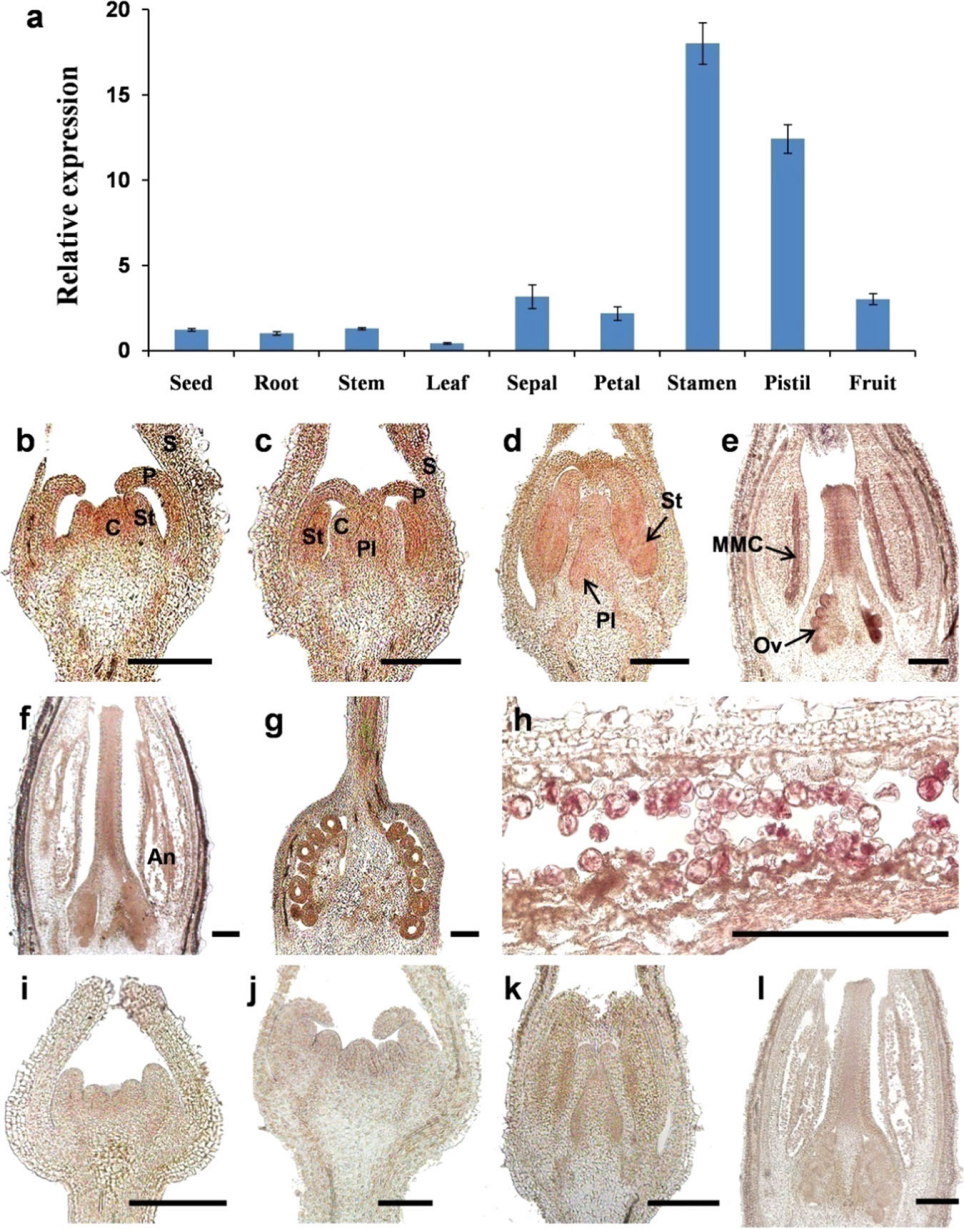
Expression analyses of *SlMYB33* in tomato. **a** qRT-PCR analyses of *SlMYB33* in different tomato tissues. The various tissues in different developmental stages included in this analysis were as follows: seeds, red ripe fruit stage; roots, stems, and leaves, four true leaf-stage plants; sepals, petals, stamens, and pistils, 5-mm-length flower buds; fruits, 7-DPA (days post anthesis) stage. Values are the mean ± SD of three biological replicates. **b**–**l** In situ hybridization of *SlMYB33* in tomato flowers at different developmental stages. **b**–**h** Longitudinal sections of flower buds hybridized with the antisense probe at stage 4 (b), stage 6 (**c**), stage 7 (**d**), stage 9 (**e**), stage 12 (**f**), and stage 14 (**g**, **h**). **i**–**l** Negative controls hybridized with the sense probe (no signals were detected). S sepal, P petal, St stamen, C carpel, Pl placenta, MMC microspore mother cell, Ov ovule, An anther. Bars = 200 μm

To address the possible functions of *SlMYB33*, we first evaluated its expression profiles in various tissues of tomato (Micro-Tom) through qRT-PCR. *SlMYB33* is mainly expressed in stamens and pistils, in which its transcript levels are at least fourfold higher than those in the seeds, roots, stems, leaves, sepals, petals, and fruits (Fig. 2a). This result was validated in the TomExpress database, and we found that the expression pattern of *SlMYB33* in TomExpress showed a similar trend to the results of the qRT-PCR analysis (Supplementary Fig. S2). We also obtained expression data for another tomato *GAMYB* gene, *SlGAMYB1*, from TomExpress. *SlGAMYB1* is highly expressed in flower buds compared with other tissues (Supplementary Fig. S2). These observations pointed to a potential role of *SlMYB33* in tomato flower development, and *SlGAMYB1* may have a similar function.

Next, we performed in situ hybridization to analyze the spatial/temporal expression of *SlMYB33* during tomato flower development in detail (Fig. 2b–l). *SlMYB33* transcripts were detected in the developing sepals, petals, stamens, carpels, and placenta primordia in early stages (stages 4 and 6, Fig. 2b, c)^24^, after which *SlMYB33* expression was restricted to the stamens and placentae (stage 7, Fig. 2d). In later developmental stages, *SlMYB33* was persistently expressed in the developing microspore mother cells (MMC), anthers, pollen grains, and ovules (stages 9–14, Fig. 2e–h). As negative controls, no signals were detected under *SlMYB33* sense probe hybridization (Fig. 2i–l). Our results suggested that *SlMYB33* may play an important role in both male and female gametophyte development in tomato.

### Knockdown of *SlMYB33* results in delayed flowering in tomato

**Fig. 3:**
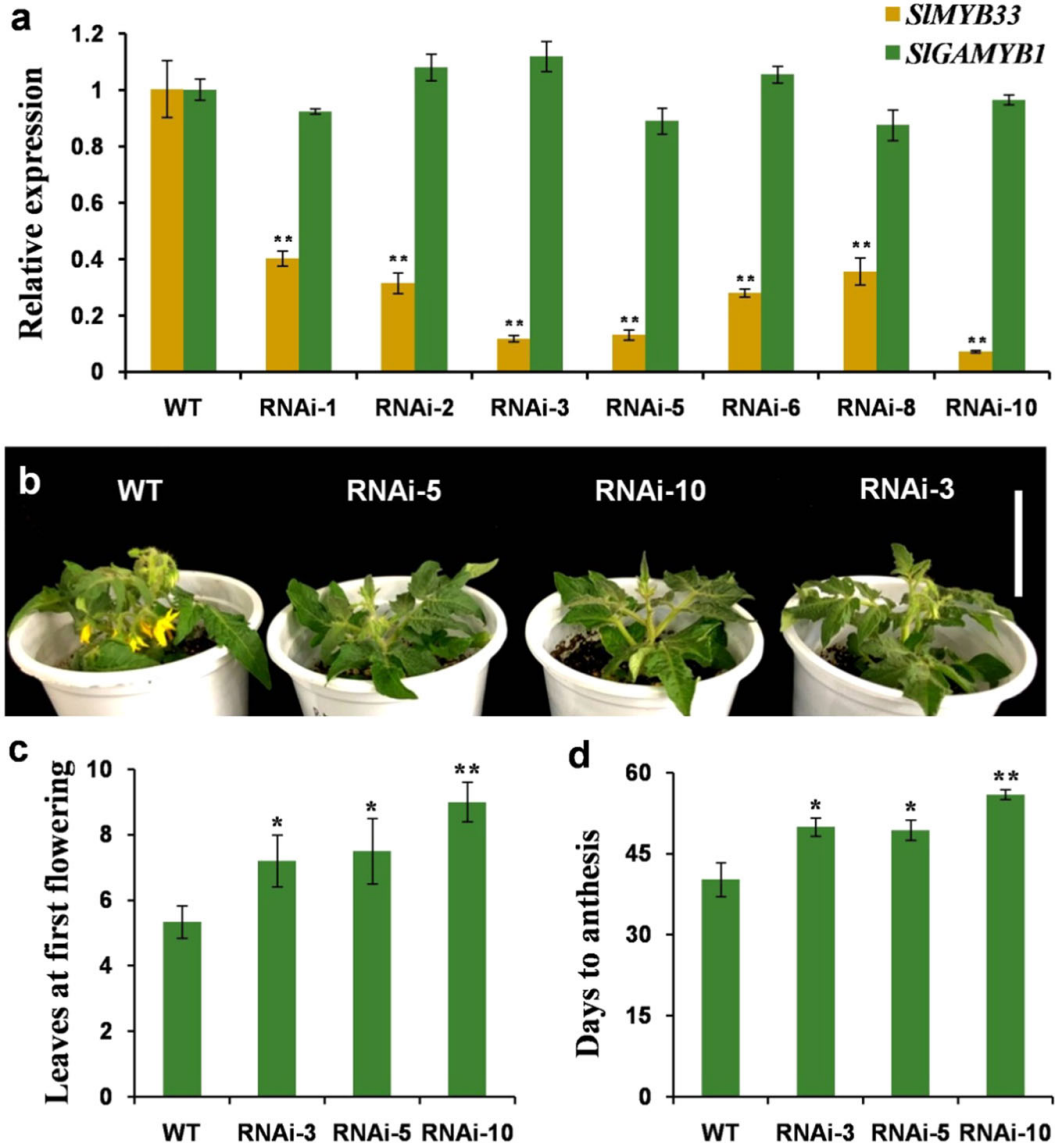
*SlMYB33*-RNAi results in delayed flowering in tomato. **a** Expression analyses of *SlMYB33* and *SlGAMYB1* in the flower buds (5-mm length) of WT plants and *SlMYB33*-RNAi lines by qRT-PCR. Each value is the mean ± SD of three biological replicates. **b** Wild-type (WT) plants and progenies of *SlMYB33*-RNAi lines. All genotypes were sown at the same time and grown under the same environmental conditions. When the flowers of the WT plants open, the RNAi lines are in the vegetative stage. Bar = 5 cm. **c** Numbers of leaves at first flowering in WT and the progenies of *SlMYB33*-RNAi lines. Each value is the mean ± SD of three biological replicates, and six plants were examined for each replicate. **d** Days to anthesis in WT and the progenies of *SlMYB33*-RNAi lines. Each value is the mean ± SD of three biological replicates, and six plants were examined for each replicate. Asterisks indicate significant differences between the RNAi lines and WT by Student’s *t* tests (*P < 0.05; **P < 0.01)

To determine the biological function of *SlMYB33*, we attempted to inhibit its expression in Micro-Tom plants using gene-silencing technology. An RNA interference (RNAi) construct under the control of the CaMV 35S promoter was generated and transformed into the tomato plants. Following PCR analysis, ten independent *SlMYB33*-RNAi lines were obtained. Among these lines, seven displayed significant reductions in *SlMYB33* transcript abundance by 60–92% in the flowers, whereas no apparent changes in *SlGAMYB1* expression were observed (Fig. 3a), suggesting that only *SlMYB33* was effectively knocked down. Due to the lower *SlMYB33* expression levels in lines 3, 5, and 10 compared with the other lines, these three lines were chosen for further analysis (Fig. 3b).

Previous studies have reported the potential effect of *GAMYB* genes on flowering^6,9,10^; therefore, the flowering time in the T_1_ generation of *SlMYB33*-RNAi lines was recorded. For each RNAi line, 18 T_1_ plants exhibiting apparent suppression of *SlMYB33* were selected for this analysis (Supplementary Fig. S3). We found that flowering was initiated in the progenies of three transgenic lines after the emergence of 7.2–9 leaves, compared with 5.3 leaves in WT (wild-type) plants (Fig. 3c). Accordingly, the first flower opened at 50–56 days after sowing in the progenies of *SlMYB33*-RNAi lines, which was later than the time of 40.3 days observed in the WT plants (Fig. 3b, d). Moreover, the extension of the flowering time in the RNAi lines was negatively correlated with *SlMYB33* expression; for instance, the lowest *SlMYB33* mRNA level in the RNAi-10 line led to the most severe late-flowering phenotype (Fig. 3c; Supplementary Fig. S3). In addition, we investigated the flowering time of T_1_ plants of three null *SlMYB33*-RNAi lines (RNAi-4, RNAi-7, and RNAi-9) that displayed no obvious repression of *SlMYB33* (Supplementary Fig. S4a). No significant changes in the numbers of leaves produced before the first flower were found in these transgenic lines compared with WT plants (Supplementary Fig. S4b). These data suggested that *SlMYB33* can promote flowering in tomato.

### Suppression of *SlMYB33* leads to aberrant pollen maturity and poor fertility

**Fig. 4:**
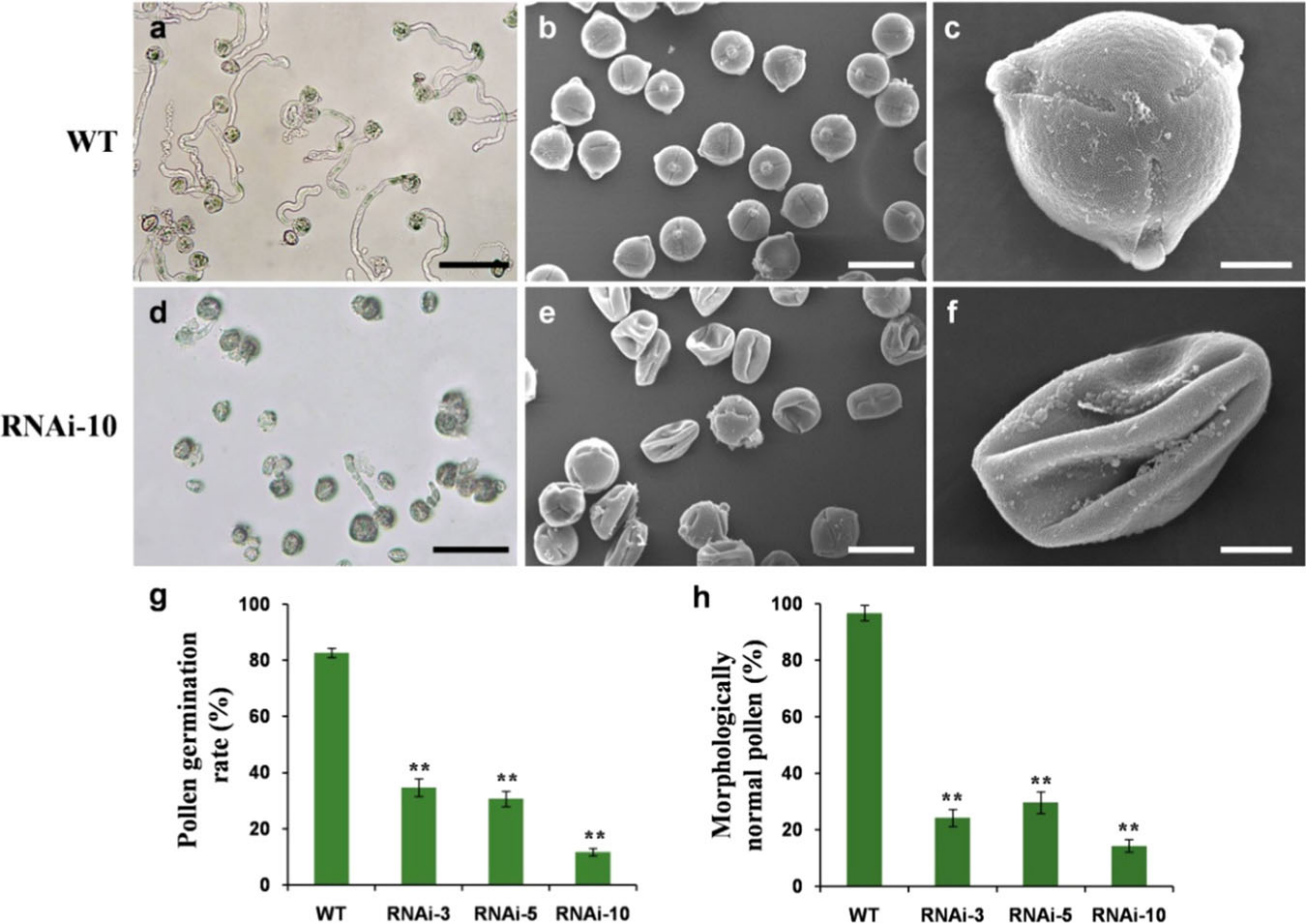
Characterization of pollen phenotypes in *SlMYB33*-RNAi lines. **a**, **d** In vitro pollen germination tests in WT (**a**) and *SlMYB33*-RNAi line 10 (**d**). Bars = 100 μm. **b**, **e** Scanning electron microscopy (SEM) images of pollen grains in WT (**b**) and RNAi-10 (**e**). Bars = 25 μm. **c**, **f** Single pollen grain morphology under SEM in WT (**c**) and RNAi-10 (**f**). Bars = 5 μm. **g**, **h** Quantification of the pollen germination rate (**g**) and percentage of morphologically normal pollen grains (**h**) in WT and different RNAi lines. Values are the means ± SD of ten biological replicates from three plants, and three to four flowers were examined in each plant. Asterisks indicate significant differences between the RNAi lines and the WT by Student’s *t* test (**P < 0.01)

To evaluate whether *SlMYB33* could affect anther and pollen development similarly to a typical *GAMYB* homolog^7,12,16,17^, the pollen phenotypes were analyzed in the *SlMYB33*-RNAi lines. As expected, a majority of the pollen grains were aberrant in the RNAi lines (Fig. 4). Here, the in vitro pollen germination assay indicated that 65–88% of the pollen grains in the *SlMYB33*-RNAi lines had lost germination ability, compared with only 17% in the WT (Fig. 4a, d, g). Further microscopic observations by SEM revealed that the pollen grains from the RNAi lines were shrunken and collapsed compared with those of the WT (Fig. 4b, c, e, f). Quantitative analyses showed that 70–86% of the transgenic pollen grains displayed an abnormal morphology, whereas this percentage was only 3% in the WT (Fig. 4h).

**Fig. 5:**
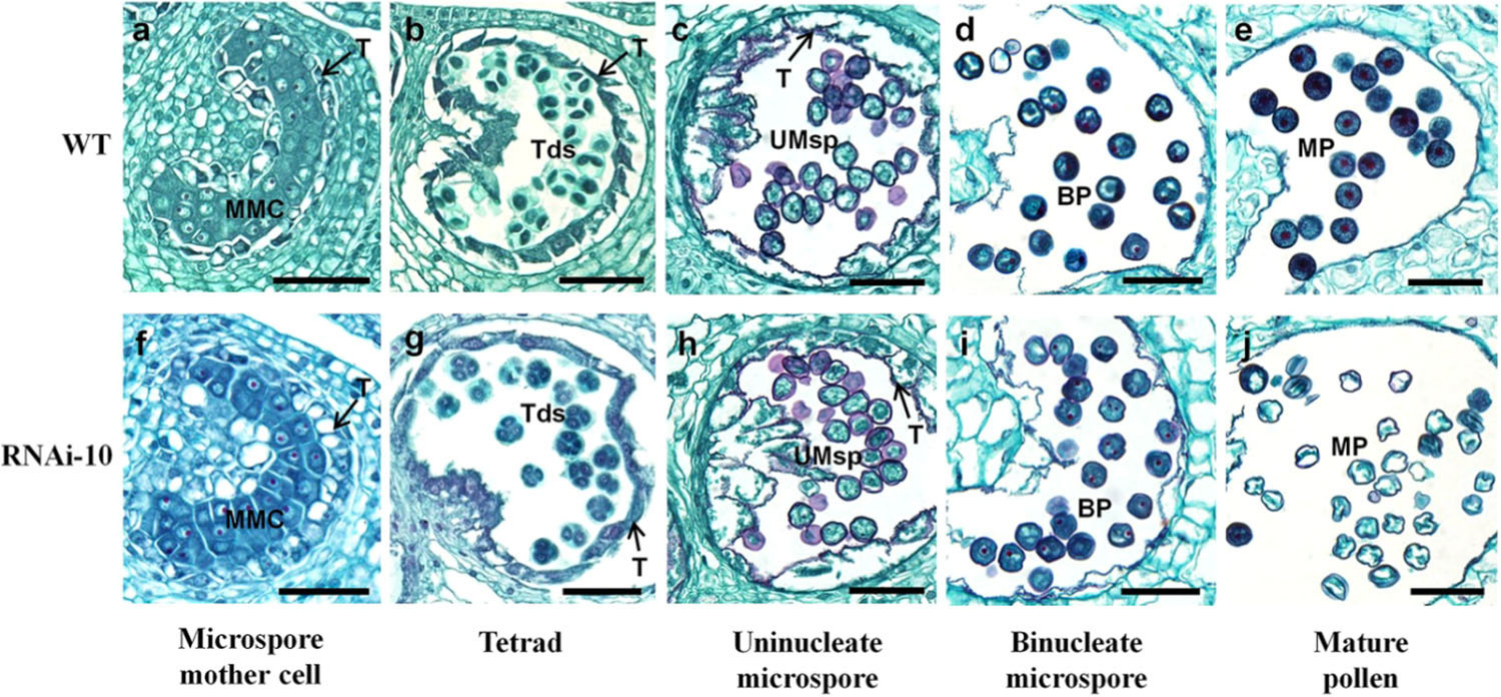
Suppression of *SlMYB33* disturbs pollen maturity. Transverse sections of flower buds at the microspore mother cell stage (**a**, **f**), tetrad stage (**b**, **g**), uninucleate microspore stage (**c**, **h**), binucleate microspore stage (**d**, **i**), and mature pollen stage (**e**, **j**) in WT and RNAi-10. MMC microspore mother cell, T tapetum, Tds tetrads, UMsp uninucleate microspore, BP binucleate pollen, MP mature pollen. Bars = 50 μm

To dissect how *SlMYB33* regulates pollen development, histological analyses of flower buds at different stages were performed in WT and RNAi plants (Fig. 5). Among the five stages of tomato pollen development^24^, no evident abnormalities were found in the microspore mother cells (MMCs), tetrads, or uninucleate microspores in the anthers of RNAi-10 plants (Fig. 5a–c) compared to those of the WT (Fig. 5f–h). During the binucleate stage, the transgenic pollen grains were slightly shrunken and irregular but showed no defects in their structures (Fig. 5d, i). However, a striking phenotype was detected at the mature stage. In contrast to the WT, most of the mature pollen grains in RNAi-10 were severely collapsed and shrunken, which may be due to the degradation of the cytoplasm (Fig. 5e, j). In addition, there was no obvious change in transgenic tapetum development (Fig. 5), which can be mediated by *GAMYB* homologs in other species such as *Arabidopsis* and rice^7,11,17^.

**Fig. 6:**
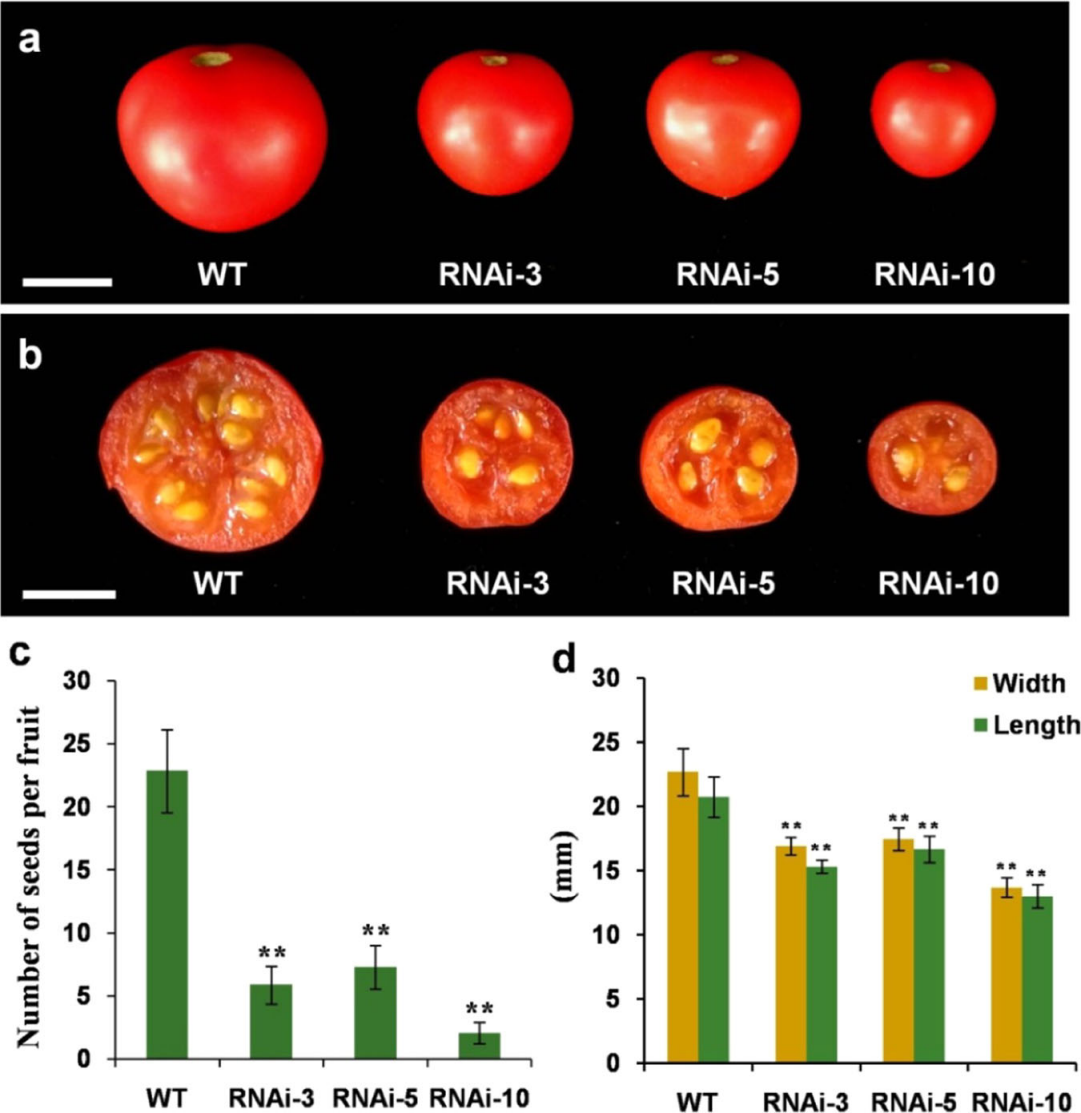
The suppression of *SlMYB33* affects the fertility and fruit size of tomato. **a** Fruits of WT and *SlMYB33*-RNAi lines. **b** Transverse sections of the fruits of WT and *SlMYB33*-RNAi lines. **c** Numbers of seeds per fruit in WT and different RNAi lines. **d** Fruit sizes in WT and different RNAi lines. Values are the means ± SD of three independent plants, and 15 fruits were examined for each plant. Asterisks indicate significant differences between the RNAi lines and the WT by Student’s *t* test (**P < 0.01). Bars = 1 cm

Along with aberrant pollen development (Figs. 4 and 5), the suppression of *SlMYB33* leads to a significant reduction in the fertility of tomato. As shown in Fig. 6, each fruit of the *SlMYB33*-RNAi lines produced only 3.1–7.3 seeds, which was a markedly lower number than 22.9 seeds recorded in the WT (Fig. 6b, c). Fruit size was also obviously decreased in the transgenic plants (fruit width of 13.7–17.5 mm, length of 13.0–16.7 mm) compared with the WT (fruit width of 22.7 mm, length of 20.8 mm), and was quantitatively related to seed number (Fig. 6a, d). Therefore, we speculated that this change in fruit size might be caused by the reduced seed number. In conclusion, the repression of *SlMYB33* disrupts pollen maturity, resulting in poor fertility accompanied by smaller fruit in tomato.

### Knockdown of *SlMYB33* restricts the expression of genes controlling flowering

**Fig. 7:**
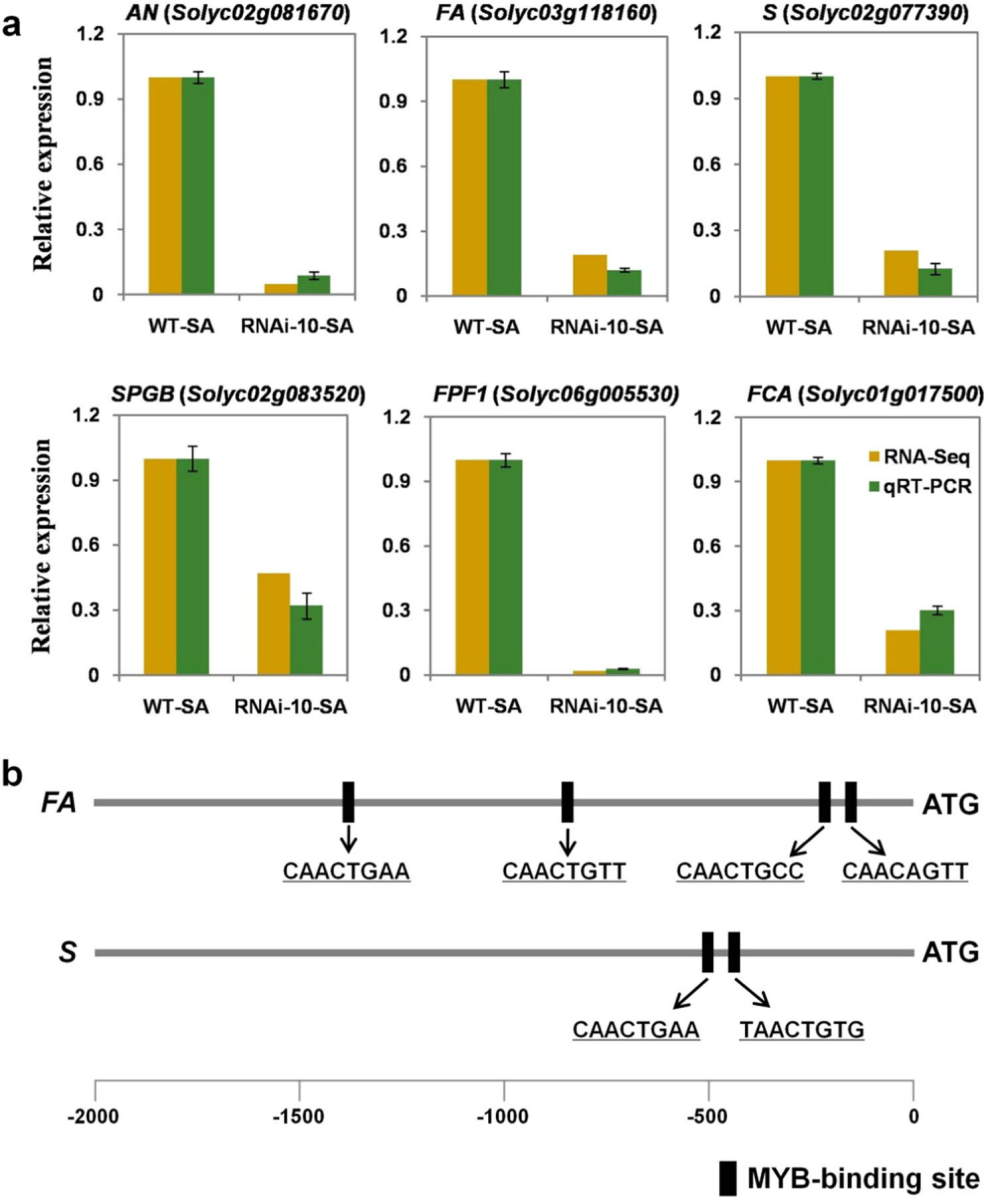
Knockdown of *SlMYB33* inhibits the expression of flowering-related genes, and *FA* and *S* act as putative candidate target genes of *SlMYB33*. **a** Expression analyses of flowering-related genes in the shoot apices of WT and RNAi-10 plants. Three independent samples used for qRT-PCR were collected at the same developmental stage as those used for RNA-Seq. Values are the mean ± SD (*n* = 3). **b** Analyses of the *cis*-acting elements in the promoters of *FA* and *S*. The 2-kb promoter sequence (before ATG) was acquired for this analysis. SA shoot apex

To identify the potential genes and molecular pathways involved in *SlMYB33*-regulated tomato flowering, transcriptome analysis was performed in shoot apices (30-day-old, not flowering) from the *SlMYB33*-knockdown line RNAi-10 and WT plants by the digital gene expression (DGE) approach^25^. Using a false discovery rate (FDR) < 0.05 and a fold change (FC) > 2 as significance cutoffs, we identified 2388 differentially expressed genes (DEGs), among which 1689 genes were upregulated, and 699 genes were downregulated in the RNAi-10 shoot apices compared with those of the WT (Supplementary Table S1, Supplementary Table S2). Through careful examination, we observed that the expression levels of several genes responsible for tomato flowering were dramatically decreased in the shoot apices of the RNAi lines, including the *ANANTHA* (*AN*), *FALSIFLORA* (*FA*), *COMPOUND INFLORESCENCE* (*S*), and *SPGB* genes, encoding the F-box protein UNUSUAL FLORAL ORGANS (UFO), the transcription factor LFY, the homeobox transcription factor WUSCHEL-HOMEOBOX9 (WOX9), and the basic region/leucine zipper (bZIP) transcription factor SP-INTERACTING G-BOX (SPGB), respectively (Fig. 7a; Supplementary Table S2)^26–28^. The tomato homologs of two genes promoting flowering in *Arabidopsis* and rice, *FLOWERING-PROMOTING FACTOR1* (*FPF1*) and *FLOWERING TIME CONTROL LOCUS A* (*FCA*), were also significantly downregulated (Fig. 7a; Supplementary Table S2)^29–34^. qRT-PCR analysis was performed for these six DEGs, and the results revealed the same expression pattern as the RNA-Seq analysis. Therefore, the *AN*, *FA*, *S*, *SPGB*, *FPF1*, and *FCA* genes may be involved in *SlMYB33*-regulated flowering in tomato. Given the ability of *GAMYB* to bind to the promoters of target genes^3,10^, we further analyzed the *cis*-acting elements in the promoters of these six DEGs. As shown in Fig. 7b, the promoters of the *FA* and *S* genes contained four and two MYB-binding sites, respectively, which were highly conserved with the 8-bp GAMYB-binding site (CAACTGTC) with a ^C^/_T_AAC core in *Arabidopsis*^10^, implying that *FA* and *S* are putative candidate target genes of *SlMYB33* in the regulation of tomato flowering.

### *SlMYB33* affects the expression of sugar metabolism genes

**Fig. 8:**
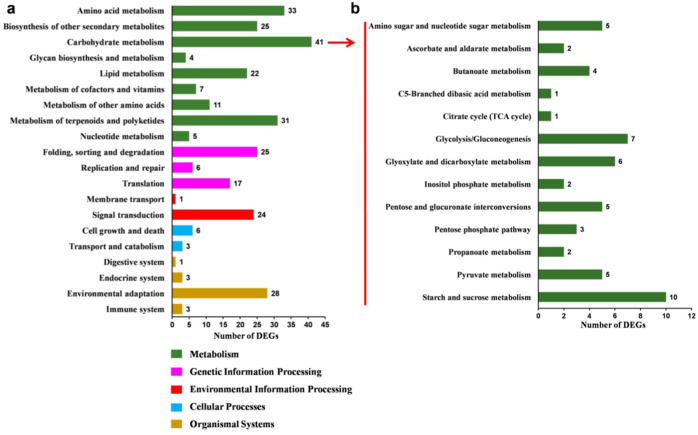
KEGG classification of DEGs in anthers between RNAi-10 and WT. **a** Functional categories of DEGs. **b** Secondary classification of the carbohydrate metabolism pathway

We also carried out RNA-Seq analysis to explore the possible gene networks through which *SlMYB33* regulates tomato pollen maturity in anthers from the transgenic RNAi-10 and WT plants. Given that gene expression differences usually occur before phenotypic changes, the anthers were harvested before the mature pollen stage (late binucleate microspore stage) and used for transcriptome analysis. Through the DGE approach, a total of 1332 DEGs, including 676 upregulated genes and 656 downregulated genes, were identified (Supplementary Tables S1 and S3). Thereafter, KEGG (Kyoto Encyclopedia of Genes and Genomes) classification was performed. The DEGs were assigned to 20 functional categories, among which “carbohydrate metabolism” was the most represented pathway, including 41 DEGs (Fig. 8a). Carbohydrate metabolism was then classified into 13 subgroups (Fig. 8b), among which starch and sucrose metabolism are essential for pollen development, especially for pollen maturity^35–39^. Further examination revealed a dramatic reduction in the transcript levels of most of the genes grouped into the starch and sucrose metabolism pathways (Table 1). For instance, *Lin7*, encoding cell wall invertase (CWIN), which hydrolyzes sucrose into glucose and fructose^39^, was significantly downregulated by 21.5-fold in the transgenic anthers compared with the WT. In addition, the expression levels of the following genes were obviously decreased: one gene encoding sucrose-phosphate synthase (SPS), which is a key regulator of sucrose synthesis, two genes that encode sucrose synthase (SUS), responsible for sucrose degradation, and one gene encoding trehalose-6-phosphate synthase (TPS), involved in the production of trehalose-6-phosphate (T6P), which acts as a signaling molecule in sensing sucrose availability and promoting starch and cell wall biosynthesis^40,41^. We further found that the transcripts of two genes encoding 6-phosphofructokinase and fructose-bisphosphate aldolase, which are two key enzymes in the glycolysis pathway, were depressed in the RNAi line. Moreover, a gene for β-glucosidase (GLU), which can release glucose from the inactive glucoside^42^, is upregulated. In addition, the gene for ADP-glucose pyrophosphorylase (AGPase), a key enzyme for starch synthesis^40,43^, exhibited a reduced mRNA level; however, a similar expression profile was detected in the β-amylase gene responsible for starch degradation. In addition, the expression of the gene (no classification) encoding sugar transporter protein 13 (SlSTP13) was remarkably inhibited (Table 1). qRT-PCR assays of the above genes showed the same expression trend as the RNA-Seq analysis (Supplementary Fig. S5). These results indicated that the silencing of *SlMYB33* disrupts tomato pollen maturity at least partly by repressing the transcription of genes related to starch and sucrose metabolism as well as sugar transport.

**Table 1:** Differentially expressed genes in the anthers of *SlMYB33*-RNAi and WT plants

Functional category	Gene ID	Gene annotation	FC	FDR
Starch and sucrose metabolism	Solyc09g010090	Cell wall invertase (Invertase 7, Lin7)	−21.53	2.76E–05
	Solyc11g045110	Sucrose-phosphate synthase (SPS)	−10.13	2.78E–02
	Solyc07g042550	Sucrose synthase (SUS3)	−4.02	4.10E–02
	Solyc12g040700	Sucrose synthase	−3.19	4.23E–05
	Solyc02g071590	Trehalose-6-phosphate synthase (TPS)	−2.07	7.14E–03
	Solyc04g015200	6-Phosphofructokinase 2	−2.06	8.53E–06
	Solyc07g065900	Fructose-bisphosphate aldolase	−3.88	2.43E–04
	Solyc08g044510	β-Glucosidase (GLU)	3.28	3.28E–02
	Solyc12g011120	ADP-glucose pyrophosphorylase (AGPase)	−3.51	1.49E–02
	Solyc09g091030	β-Amylase 1	−3.10	3.37E–02
	Solyc03g005150	Sugar transporter protein 13 (SlSTP13)	−4.15	4.47E–02
Pentose and glucuronate interconversions	Solyc06g009190	Pectinesterase (PE)	2.47	6.27E–05
	Solyc05g047590	Pectinesterase (PE)	2.02	3.56E–02
	Solyc03g111690	Pectate lyase (PL)	2.08	2.56E–02
	Solyc06g068040	Polygalacturonase (PG)	−3.28	4.95E–03

Furthermore, we observed remarkable expression differences in several genes (belonging to the “pentose and glucuronate interconversions” pathway) controlling cell wall degradation in transgenic RNAi anthers compared with those of the WT. Here, two pectinesterase (PE) genes and one pectate lyase (PL) gene displayed upregulation, whereas one polygalacturonase (PG) gene showed downregulation (Table 1; Supplementary Fig. S5), revealing the possible involvement of these genes in pollen collapse in *SlMYB33*-RNAi plants.

**Fig. 9:**
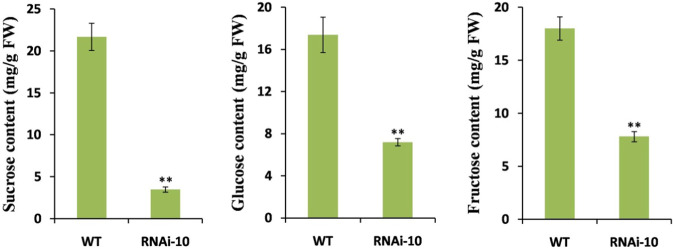
Sucrose, glucose, and fructose concentrations in WT and RNAi-10 anthers at the mature pollen stage. Values are means ± SD of three biological replicates. Asterisks indicate significant differences between RNAi-10 and WT plants by Student’s *t* test (**P < 0.01)

We then performed metabolite analysis to examine whether the changes in the expression of genes related to sugar metabolism could influence the contents of carbohydrates. As expected, we found that the knockdown of *SlMYB33* significantly decreased the sucrose content by 6.2-fold in anthers at the mature pollen stage, and the concentrations of glucose and fructose also declined in the RNAi-10 plants (Fig. 9).

## Discussion

### *GAMYB* functions in the regulation of flowering

Our results suggested that the knockdown of *SlMYB33* delays tomato flowering (Fig. 3). However, there are differing reports about the roles of *GAMYBs* in the regulation of flowering. Overexpression of miR159 in *Arabidopsis* ecotype *Landsberg erecta* causes late flowering through the downregulation of the target *AtMYB33* under short days^6^; however, in *Columbia*, the overexpression of miR159, repressing *AtMYB101*, does not alter flowering time under long days^8^. It has also been reported that the loss of function of *GAMYBs* in *Arabidopsis* (*Columbia* background) and rice does not affect flowering^11,12^; moreover, the transcriptomes of the shoot apices are almost identical between WT and *myb33 myb65* mutant in *Arabidopsis*^11^. Nevertheless, in gloxinia, the overexpression or repression of miR159 causes the downregulation or upregulation of *GAMYB*, leading to delayed or early flowering, respectively^13^. In contrast, a recent report demonstrated that transgenic tobacco plants with greater *GAMYB* levels exhibit a late-flowering phenotype^14^. These observations indicated that the functions of *GAMYBs* in flowering appear to be determined by a complex mechanism, which may be affected by differences in species or ecotypes. This is the first time that the direct downregulation of *GAMYB* has been shown to result in late flowering, providing novel evidence of the diversity of *GAMYB* functions in flowering.

### *SlMYB33* shows conserved and different roles in pollen development compared with its homologs

*GAMYBs* have been verified to positively regulate stamen development. Loss of function of *GAMYBs* leads to defects in stamen, anther, and pollen development, resulting in male sterility in *Arabidopsis* and rice^7,12,16,17^. In our study, expression analyses showed that tomato *SlMYB33* is highly expressed in staminate organs (Fig. 2). The knockdown of *SlMYB33* results in aberrant and inviable pollen grains (Fig. 4) and ultimately poor fertility (Fig. 6). Although fruit size also decreases in *SlMYB33*-RNAi lines, this may be a consequence of the reduced seed number in fruits (Fig. 6). These data supported the notion that *SlMYB33* exhibits a conserved function in the promotion of pollen development.

Moreover, *Arabidopsis myb33 myb65* and rice *gamyb* mutants show abnormal pollen development, owing to the loss of programmed cell death (PCD) and consequent endless hypertrophy of the tapetum^7,11,17^, demonstrating the importance of *GAMYB* in tapetum degradation. However, we did not find any obvious change in tapetum morphology at various developmental stages in *SlMYB33*-RNAi anthers compared with those of the WT, while the abortion of transgenic pollen grains was caused by damaged pollen maturity (Fig. 5), indicating that *SlMYB33* plays a key role in tomato pollen maturity, rather than tapetum development. Therefore, despite the positive effect of both *SlMYB33* and its homologs on pollen development, their regulatory mechanisms are different.

### The potential effect of BR–GA crosstalk in phenotypes caused by the knockdown of *SlMYB33*

The tomato cultivar Micro-Tom was used as the studied plant material in this work. This genotype is a brassinosteroid (BR)-deficient mutant that exhibits a very dwarf phenotype with small fruits, but with no effect on flowers^44^. Further study suggested that BR and GA synergistically mediate vegetative growth in Micro-Tom^45^. BRs and GAs are two groups of growth-promoting phytohormones that can interact during many developmental processes^46–49^. BR induces GA biosynthesis to regulate plant growth, affecting processes such as cell elongation in rice^50^ and seed germination, hypocotyl elongation, and flowering in *Arabidopsis*^51^. Domagalska et al.^52^ also reported that the promotion effect of BR on flowering depends on the presence and concentration of GA in *Arabidopsis*^52^. On the other hand, GA can regulate plant growth by modulating BR biosynthesis and signaling. *SPINDLY* (*SPY*), a negative regulator of GA signaling, inhibits BR biosynthesis to mediate lamina joint bending^53^, whereas *GERMOSTATIN RESISTANCE LOCUS 1* (*GSR1*), a positive regulator of GA signaling, stimulates BR biosynthesis to control root and leaf development and fertility in rice^54^. Moreover, DELLA proteins, which are the key repressors of GA signaling, interact with the positive regulators of BR signaling BRASSINAZOLE-RESISTANT1 (BZR1)/*BRI1*-EMS-SUPPRESSOR1 (BES1) and repress their activities. Therefore, GA can release the DELLA-modulated inhibition of BZR1/BES1 to induce BR responses and plant growth^55–57^.

In this work, phenotypic differences were observed between *SlMYB33*-RNAi lines and WT plants of the same genetic background (Micro-Tom), indicating that the phenotypes were caused by the silencing of *SlMYB33*, rather than BR deficiency in Micro-Tom. However, given the general feedback regulation of GA production by GA signaling^58^, together with the complexity of BR–GA interaction, it remains possible that the knockdown of *SlMYB33* affects the crosstalk between BR and GA at the biosynthesis level and/or the signaling level, resulting in late flowering and abnormal pollen development.

### Flowering-related genes are candidate genes regulated by *SlMYB33*

Our data revealed the important finding that the knockdown of *SlMYB33* affects the expression of some genes controlling flowering, including *FA*, *AN*, *S*, *SPGB*, *FPF1*, and *FCA* (Fig. 7a). *FA* is an ortholog of *Arabidopsis LFY*, which is responsible for flower initiation^26^. Previous studies have demonstrated that the *LFY* can be activated by the application of GA, while *GAMYBs* influence flowering by regulating the GA responsiveness of the *LFY* promoter in *Arabidopsis*^10,59^. Likewise, *FA* can mediate tomato flowering, as demonstrated by a late-flowering phenotype with an increased number of leaves before the first inflorescence in the *fa* mutant^26^. *AN* encodes an F-box homolog of *Arabidopsis* UFO, while the *S* gene encodes a WOX9 transcription factor. *S* and *AN* are sequentially expressed during the phase transition from the inflorescence meristem to the floral meristem, and play overlapping roles in inflorescence architecture as well as floral identity. A mutation in either *AN* or *S* delays tomato flowering, leading to highly branched inflorescences^21,27^. Genetic interaction analysis shows that *AN* functions downstream of *FA*, demonstrating the crucial function of *FA* in flowering^27^. Here, our work revealed that the silencing of *SlMYB33* results in late flowering (Fig. 3), and significantly reduces the transcript levels of *FA*, *AN*, and *S* (Fig. 7a). In addition, the promoters of the *FA* and *S* genes contain putative GAMYB-binding sites (Fig. 7b). Based on these findings, we speculated that *SlMYB33* controls flowering partly via the transcriptional activation of the *FA*–*AN* pathway and the *S* gene. Between the *FA* and *S* candidates, the former is most likely a downstream target of *SlMYB33*, as indicated by the homology of *SlMYB33* and *FA* to *Arabidopsis AtMYB33* and *LFY*, respectively (Fig. 1; Supplementary Fig. S1)^26^, while *AtMYB33* functions upstream of *LFY*^10^.

### *SlMYB33* probably modulates pollen maturity by regulating the expression of sugar metabolism genes

Our work also highlighted a potential role of *SlMYB33* in mediating the expression of genes related to sugar metabolism (Fig. 8; Table 1), which is necessary for pollen development^36,37^. For pollen to be viable, the import of sufficient nutrients is essential to support a series of physiological activities. CWIN hydrolyzes sucrose into glucose and fructose, which are then taken up into pollen by hexose transporters following an apoplasmic pathway^39,40,60^. Previous studies have reported the crucial role of *CWIN* in pollen development. The downregulation of tobacco *CWIN* results in inviable pollen because of the loss of starch and cell wall integrity^61^. In addition, pollen sterility is attributable to a reduction in the expression of *CWIN* together with hexose transporter genes under cold stress in rice^62^. Accordingly, we identified a dramatic decrease in the expression of the tomato *CWIN* ortholog *Lin7* (*SlCWIN3*)^40^, in *SlMYB33*-RNAi anthers (Table 1). *Lin7* has been reported to be predominately expressed in tomato anthers^63^; however, its biological function is still elusive. Chen et al.^35^ speculated that *Lin7* potentially plays a role in postmeiotic pollen development, while our data revealed a possible effect of *Lin7* on pollen maturity in tomato, suggesting that the function of *CWIN* homologs in pollen development may be conserved. Moreover, one *SPS* and two *SUS* genes were obviously downregulated in transgenic anthers (Table 1). SPS is a key enzyme for sucrose synthesis, whereas SUS contributes to sucrose cleavage to generate UDP-glucose and fructose^40^. Likewise, *SlSTP13*, a sugar transporter gene, was inhibited in the transgenic lines (Table 1). STPs serve as hexose/H^+^ symporters and contribute to the transport of glucose and fructose from the cell wall to the cytoplasm^64^.

Starch is highly important as an energy source in the microsporangium, and is essential for pollen ontogeny^38^. AGPase catalyzes the first key step of starch biosynthesis^65^^,66^. Strikingly, SPS activity is positively correlated with starch accumulation^67^. Therefore, the downregulation of *AGPase* and *SPS* (Table 1) indicated that the starch concentration might be decreased in *SlMYB33*-RNAi anthers. Moreover, a reduced mRNA level of one gene encoding β-amylase was observed (Table 1); this enzyme is an exoenzyme responsible for starch cleavage to release maltose molecules^38^, suggesting that starch degradation is also influenced.

In conclusion, the abnormal pollen maturity observed in *SlMYB33*-knockdown plants might be caused at least in part by the restriction of sucrose and starch metabolism and sugar transport, leading to a lack of nutrient reserves in pollen grains. The decreased sucrose, glucose, and fructose contents detected in RNAi-10 anthers at the mature pollen stage (Fig. 9) provided further evidence to support this proposition.

### The possible role of miR159 in *SlMYB33*-regulated flowering and pollen development

Through BLAST analysis with mature miR159 sequences from *Arabidopsis* in miRbase (http://www.mirbase.org)^6,68^, we found two miR159 members in tomato: miR159a (MIMAT0009141) and miR159b (MIMAT0042036) (Supplementary Fig. S6a). A binding site was then detected between *SlMYB33* and miR159a/b (Supplementary Fig. S6b), suggesting that *SlMYB33* may act as the target of miR159a and miR159b.

Previous reports have indicated that miR159 functions in a feedback-regulatory loop with *GAMYB* in *Arabidopsis* in which miR159 directs the downregulation of *GAMYB* activity and is then compensatorily upregulated by *GAMYB*^6^. Here, we performed comparative expression analyses of miR159a and miR159b between *SlMYB33*-knockdown and WT plants; however, no significant changes were observed in either the shoot apices or anthers (Supplementary Fig. S6c), implying that there may be no feedback regulation of miR159 by *SlMYB33* in tomato.

Based on the above observations and the high conservation of the miR159 family^5^, we predicted that *SlMYB33*-regulated flowering and pollen development may be controlled by miR159 (a/b) in tomato, but without the involvement of a feedback-regulatory mechanism.

## Materials and methods

### Plant materials and growth conditions

Tomato (*Solanum lycopersicum* L. cv Micro-Tom) seeds were germinated in a petri dish at 28 °C in the dark for 3 days, and the seedlings were then cultured in a growth room under a 16-h/8-h photoperiod with day/night temperatures of 25 °C/18 °C. Water management and pest control were performed according to standard practices.

### *SlMYB33* cloning, sequence alignment, and phylogenetic analysis

Total RNA was extracted from tomato flower buds using an RNA extraction kit (TaKaRa, Kusatsu, Japan), and cDNA was synthesized using the PrimeScript RT reagent kit (TaKaRa). The coding sequence (CDS) of *SlMYB33* was amplified by PCR using gene-specific primers (Supplementary Table S4). The protein domain structure of SlMYB33 was analyzed using the software DOG 2.0.

The amino acid sequences of related GAMYB proteins in various species were obtained from the Solanaceae Genomics Network (http://www.solgenomics.net), National Center for Biotechnology Information (http://www.ncbi.nlm.nih.gov), or Phytozome v12.1 (https://phytozome.jgi.doe.gov/pz/portal.html) database. Then, multiple-sequence alignment was carried out using MEGA5 software^69^, and boxes highlighting conserved sequences were drawn using the online software BoxShade (http://www.ch.embnet.org/software/BOX_form.html). The phylogenetic analysis was conducted via the neighbor-joining method^70^ with MEGA5, and bootstrapping was performed with 1000 replications.

### qRT-PCR analysis

For the gene expression assay, total RNA extraction and cDNA synthesis were performed using an RNA extraction kit (TaKaRa, Kusatsu, Japan) and a PrimeScript RT reagent kit (TaKaRa), respectively. Then, quantitative real-time reverse transcription polymerase chain reaction (qRT-PCR) was carried out using the SYBR Premix Ex Taq kit (TaKaRa) with an Applied Biosystems 7500 real-time PCR system (Applied Biosystems, CA, United States). The tomato *EF-1α* gene served as the reference gene^71^. Each qRT-PCR assay was repeated with three biological samples. The relative expression levels of genes were calculated using the 2^–ΔΔCt^ method.

For the mature miRNA159 expression analysis, the cDNA was generated through reverse transcription from the total RNA by using the miRcute miRNA First-Strand cDNA Synthesis Kit (TIANGEN, Beijing, China). The qRT-PCR analysis was performed using an miRNA qPCR Detection Kit (SYBR Green) (TIANGEN). The tomato *U6* small nuclear RNA gene was used as an internal control. The gene-specific primers used in these procedures are listed in Supplementary Table S4.

### In situ hybridization

Developing tomato flower buds at various developmental stages^24^ were collected, fixed, embedded, sectioned, and subjected to in situ hybridization as described previously^72^. The sense and antisense probes for *SlMYB33* were generated using SP6 or T7 RNA polymerase, respectively. The primers used for the synthesis of the probes are listed in Supplementary Table S4.

### Construction of the RNAi vector and tomato transformation

A 496-bp fragment of the *SlMYB33* coding sequence was amplified to produce the sense strand using primers containing *Spe* I and *Bam*H I sites and the antisense strand using primers containing *Asc* I and *Swa* I sites. The two amplified fragments were cloned into the pFGC1008 vector in the reverse orientation. Then, the resulting *SlMYB33-*RNAi vector was transformed into tomato as described by Chen et al.^35^. The positive plants were verified through PCR testing. After self-crossing, the seeds of the T_0_ generation were harvested and then sown in the soil matrix together with those of the WT. The seedlings of the T_1_ lines and WT were cultured in a growth room under the same environmental conditions. To avoid the impact of external factors such as tissue culture procedures and antibiotics on phenotypic identification, the positive transformants of the T_1_ generation were selected by PCR examination rather than by antibiotic screening. The specific primers used for RNAi construct generation and the identification of transformants are listed in Supplementary Table S4.

### Pollen germination

Pollen germination tests were performed according to Carrera et al.^73^. Briefly, pollen grains were collected and deposited on glass slides with germination medium (pH 5.8) containing 0.292 M sucrose, 1.27 mM Ca(NO_3_)_2_, 1.62 mM H_3_BO_3_, 1 mM KH_2_PO_4_, and 0.6% agarose. After incubation at 25 °C in the dark for 2 h, the germinated pollen grains were observed and counted using a microscope.

### Scanning electron microscopy (SEM)

The mature anthers were collected and fixed overnight in 4% glutaraldehyde at 4 °C and washed with 0.1 M phosphate-buffered saline (PBS, pH 6.8) four times. The samples were dehydrated through an ethanol series (10%, 30%, 50%, 70%, 80%, 90%, and 100%), critical point dried using a desiccator, and coated with gold–palladium in an ion coater (Eiko IB5, Tokyo, Japan). Digital images of pollen morphology were observed using a scanning electron microscope (Hitachi S-4800, Tokyo, Japan) with an accelerating voltage of 2 kV^74^.

### Histology

Samples of flower buds containing various developmental stages of pollen^24,35^ were fixed, embedded, transversely sectioned (5-μm thick), dewaxed, and stained as previously described^75^. The sections were stained with 1% sarranine and 0.5% Fast Green solution and viewed under a light microscope (Olympus BX51, Tokyo, Japan) equipped with a digital camera (Olympus DP72, Tokyo, Japan).

### Transcriptome analysis

Total RNAs from the shoot apices and anthers at specified stages were extracted with an RNA kit (TaKaRa) and used for transcriptome analysis. Digital gene expression (DGE) libraries were constructed as described previously^19^. Three biological replicates were included in each library. RNA sequencing was performed on the Illumina HiSeq 4000 platform using paired-end technology by Majorbio Corporation (Shanghai, China). Bioinformatics analysis of the DGE data was carried out using the free online platform of the Majorbio Cloud Platform (www.majorbio.com). The differentially expressed genes (DEGs) were identified using the DESeq2 tool^76^ with an FDR (false discovery rate) < 0.05 and a fold change > 2. The KEGG (Kyoto Encyclopedia of Genes and Genomes) classification of DGEs was conducted with KOBAS 2.0 software^77^.

### Analysis of the promoters of target genes

The 2-kb promoter sequences (before ATG) of the target genes were acquired, and the *cis*-acting elements were analyzed using the online database PlantCARE (http://www.bioinformatics.psb.ugent.be/webtools/plantcare/html/).

### Determination of sucrose, glucose, and fructose contents

Anthers at the mature pollen stage were collected and immediately frozen in liquid nitrogen. The concentrations of sucrose, glucose, and fructose were measured by HPLC (Shimadzu LC-30A, Kyoto, Japan) following Chen’s method^35^, and sucrose (CAS: 57-50-1), D-( + )-glucose (CAS: 50-99-7), and D-(-)-fructose (CAS: 57-48-7, Sigma, USA) were used as the corresponding standards.

### Statistical analysis

Data analyses were performed using Excel 2010. The significance of differences between the control and experiment groups was assayed using two-tailed Student’s *t* tests with SPSS (Statistical Product and Service Solutions) 23.0. The threshold values corresponding to *P* < 0.05 and *P* < 0.01 were indicated as * and **, respectively.

### Accession numbers

The accession numbers of the GAMYB orthologs from various species used in this study are as follows: *Arabidopsis* MYB33 (At5g06100), MYB65 (At3g11440), MYB101 (At2g32460), *Capsicum annuum* GAMYB1 (CA01g13490), GAMYB2 (CA06g22300), *Coffea canephora* MYB65 (Cc01_g07440), *Cucumis melo* GAMYB (XP_008456639), *Cucumis sativus* GAMYB1 (Csa009014), GAMYB2 (Csa019830), GAMYB3 (Csa013555), *Glycine max* GAMYB1 (HM447241), GAMYB2 (HM447242), *Gossypium raimondii* GAMYB-like (Gorai.009G301100), *Hordeum vulgare* GAMYB (AAG22863), *Lolium temulentum* GAMYB (AAD31395), *Malus domestica* GAMYB-like (MDP0000147309), *Manihot esculenta* GAMYB-like (XP_021598631), *Oryza sativa* GAMYB (CAA67000), *Petunia hybrida* MYB33a (Peaxi162Scf00526g00820), MYB33b (Peaxi162Scf00342g00113), *Populus trichocarpa* GAMYB (XP_002313492), *Prunus persica* GAMYB (Prupe.2G050100), *Ricinus communis* GAMYB (XP_015577302), SlGAMYB1 (Solyc01g009070), SlMYB33 (Solyc06g073640), *Solanum pennellii* GAMYB (Sopen01g004560), GAMYB-like (Sopen06g030050), *Solanum tuberosum* GAMYB1 (PGSC0003DMG400022689), *Solanum tuberosum* GAMYB2 (PGSC0003DMG400005918), *Sorghum bicolor* GAMYB (Sobic.003G331100), *Vitis vinifera* GAMYB (XP_010651548), and *Zea mays* GAMYB (AIW47221).

## Supplementary information

Supplementary Figure S1-S6 and Table S1 and S4

Supplementary Table S2

Supplementary Table S3
