# Supplementary material for: The GAMYB-like gene SlMYB33 mediates flowering and pollen development in tomato
Source: Hortic Res. 2020 Sep 1;7:133. doi: 10.1038/s41438-020-00366-1 (PMC7459326; doi:10.1038/s41438-020-00366-1)
Supplement: Supplementary file 1 — Supplementary Figure S1-S6 and Table S1 and S4 [file 41438_2020_366_MOESM1_ESM.docx]

**Title: The *GAMYB-like* gene *SlMYB33* mediates flowering and pollen development in tomato**

Yan Zhang^1 *^, Bo Zhang^1 *^, Tongwen Yang^1^, Jie Zhang^1^, Bin Liu^2^, Xiangqiang Zhan^1^, Yan Liang^1^

**Supplementary Data**

Supplementary Fig. S1. Sequence alignment of the amino acid residues of SlMYB33 and its homologs in different species.

Supplementary Fig. S2. Expression data of *SlMYB33* and *SlGAMBY1* in different tissues of Micro-Tom from the TomExpress database.

Supplementary Fig. S3. qRT-PCR analyses of *SlMYB33* in T_1_ plants of *SlMYB33*-RNAi lines compared with the WT.

Supplementary Fig. S4. The null *SlMYB33*-RNAi lines has no effect on tomato flowering time.

Supplementary Fig. S5. qRT-PCR verification of sugar metabolism genes in the anthers from WT and RNAi-10 plants.

Supplementary Fig. S6. Tomato miR159 sequences and expression analyses in *SlMYB33*-RNAi plants compared with the WT.

Supplementary Table S1. Summary of the transcriptome data.

Supplementary Table S2. List of differentially expressed genes between *SlMYB33*-RNAi and WT shoot apices.

Supplementary Table S3. List of differentially expressed genes between *SlMYB33*-RNAi and WT anthers.

Supplementary Table S4. List of primers used in this study.

**
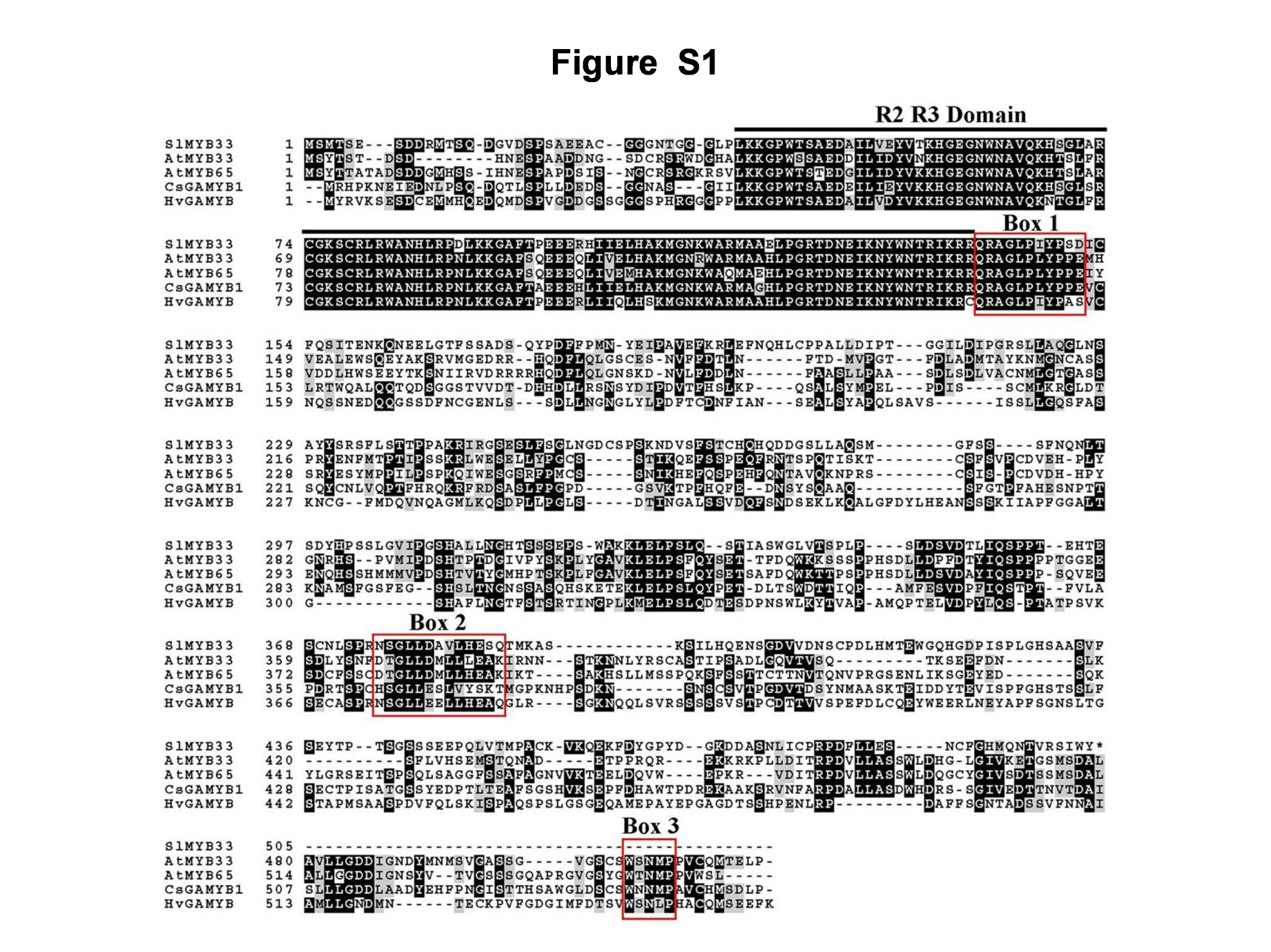
**

**Supplementary** **Fig. S1. Sequence alignment of the amino acid residues of SlMYB33 and its homologs in different species.** The conserved R2R3 repeat domain and Box 1, 2, 3 are shown in the black line and red boxes, respectively. Sl, *Solanum lycopersicum*; At, *Arabidopsis thaliana*; Cs, *Cucumis sativus*; Hv, *Hordeum vulgare*.

**
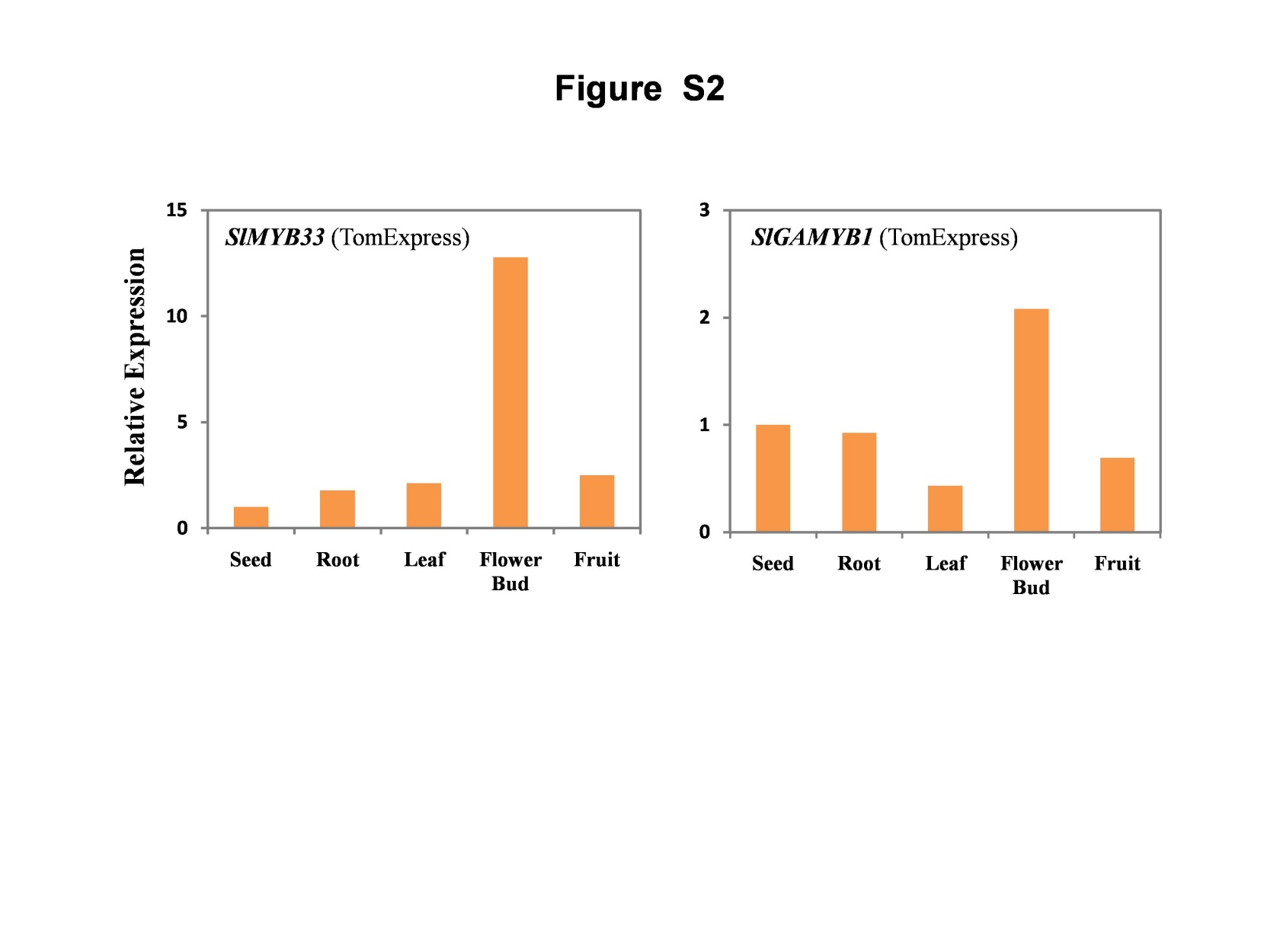
**

**Supplementary** **Fig. S2. Expression data of *SlMYB33* and *SlGAMBY1* in different tissues of Micro-Tom from the TomExpress database.** The seeds of red ripe fruits (44 DPA), flower buds of 3 mm length, and fruits of 4 DPA were used for this analysis. The various tissues were selected at the similar developmental stage with those for qRT-PCR analysis.

**
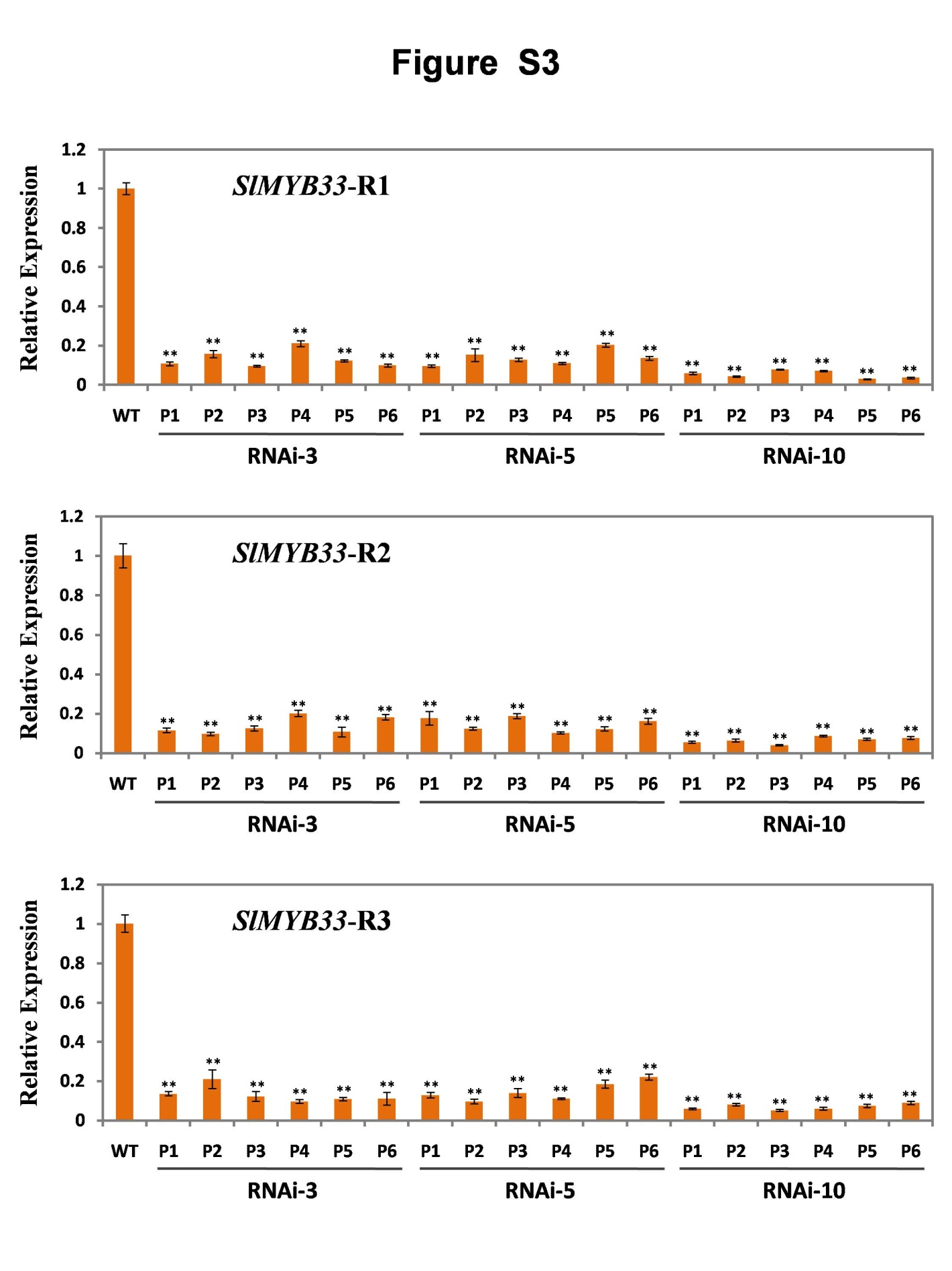
**

**Supplementary** **Fig. S3. qRT-PCR analyses of *SlMYB33* in T_1_ plants of *SlMYB33*-RNAi lines compared with the WT.** The flower buds (5 mm-length) from these plants were collected and used for this experiment. 18 T_1_ plants from three biological replicates for each RNAi line exhibited apparent suppression of *SlMYB33*, and were then chosen for analysis of flowering time. Each value is the mean ± SD of three biological replicates. Asterisks indicate significant differences between RNAi plants and WT by Student’s *t*-tests (** *P* < 0.01). R, replicate; P, plant.

**
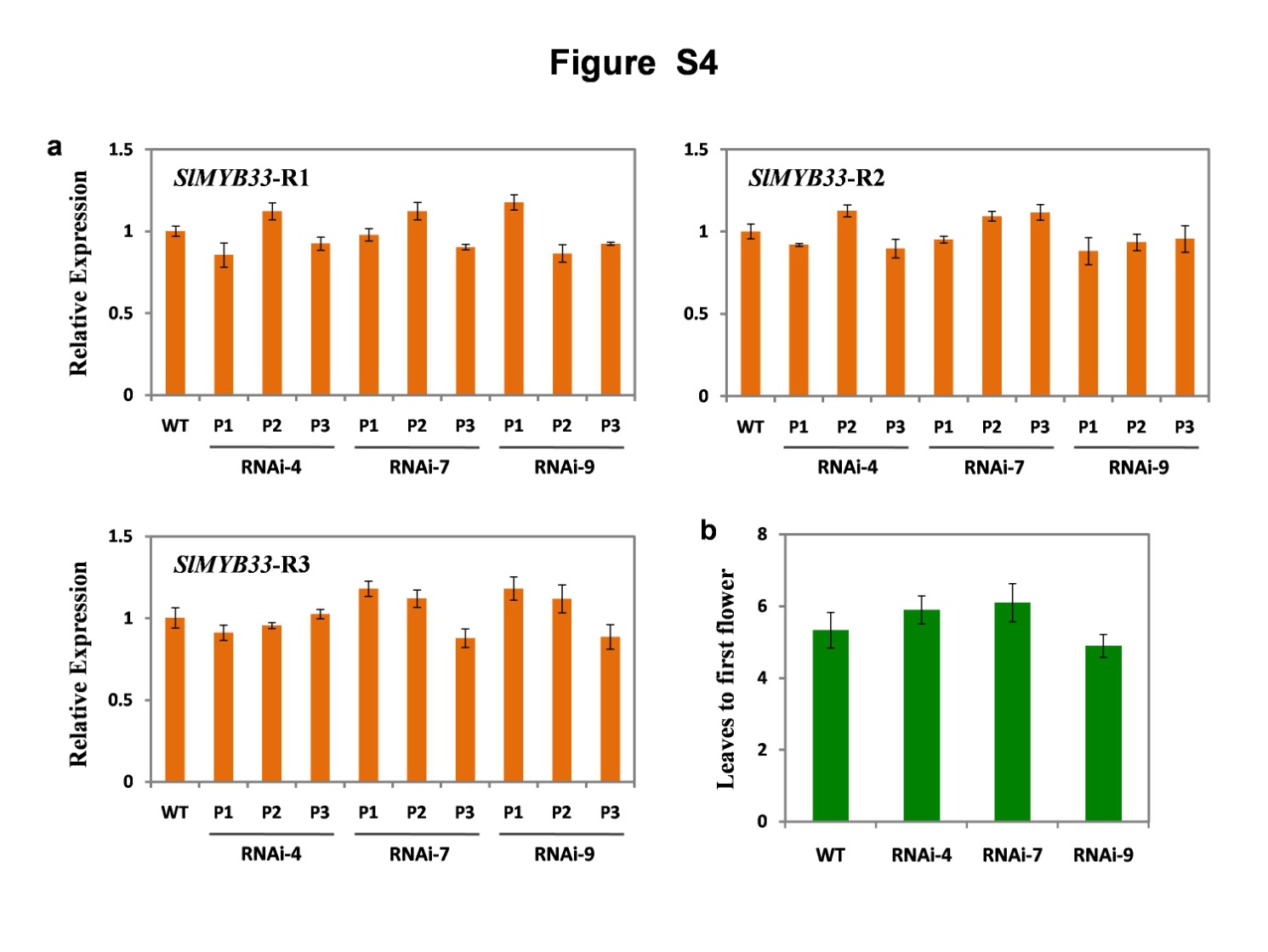
**

**Supplementary** **Fig. S4. The null *SlMYB33*-RNAi lines has no effect on tomato flowering time.** (a) qRT-PCR analyses of *SlMYB33* in flower buds (5 mm-length) of WT and T_1_ plants of null *SlMYB33*-RNAi lines. 9 T_1_ plants from three biological replicates for each RNAi line displayed no obvious repression of *SlMYB33*, and were then selected for investigation of flowering time. Each value is the mean ± SD of three biological replicates. R, replicate; P, plant. (b) Numbers of leaves to the first flower in WT and T_1_ plants of null *SlMYB33*-RNAi lines. Each value is the mean ± SD of three biological replicates, 3 plants were examined for each replicate.

**
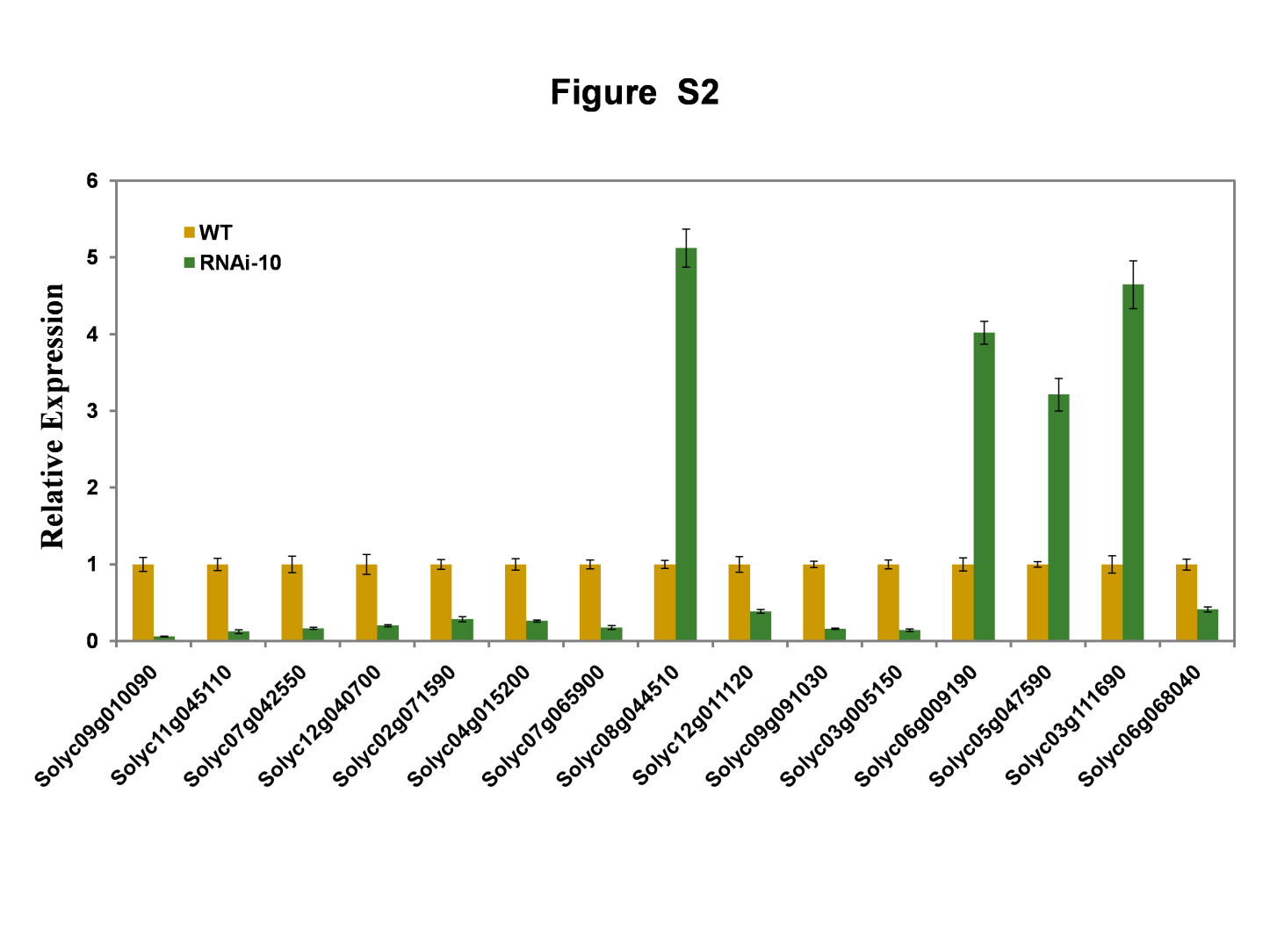
**

**Supplementary** **Fig. S5. qRT-PCR verification of sugar metabolism genes in the anthers from WT and RNAi-10 plants.** Three independently samples were collected at the same developmental stage as those for RNA-Seq. Values are the mean ± SD (n = 3).

**
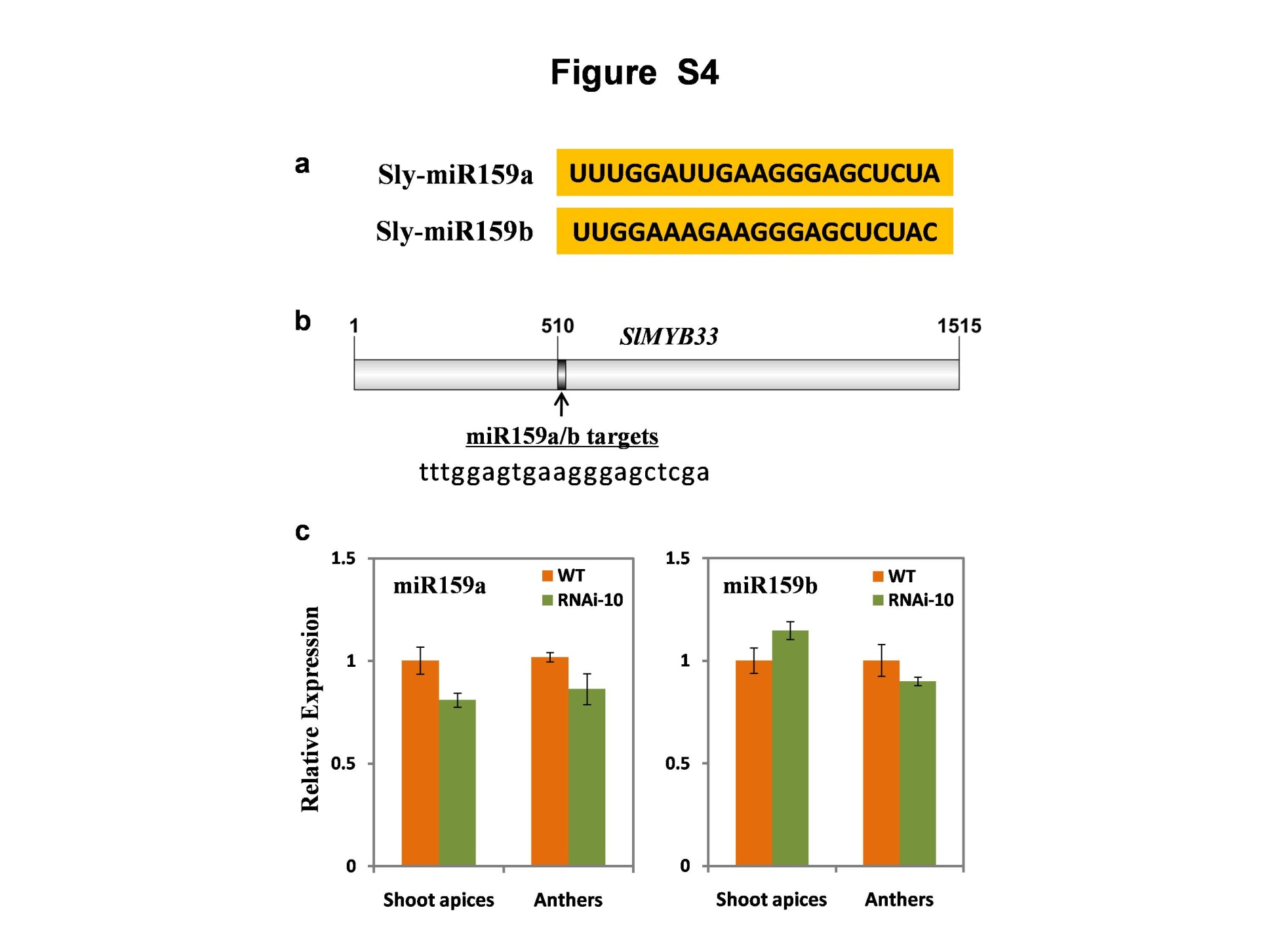
**

**Supplementary Fig. S6. Tomato miR159 sequences and expression analyses in *SlMYB33*-RNAi plants compared with the WT.** (a) Sequences of mature miR159a and miR159b in tomato. Sly, *Solanum lycopersicum*. (b) MiR159 binding site of *SlMYB33* gene. (c) qRT-PCR detection of miR159a and miR159b in shoot apices and anthers of WT and RNAi-10 plants. The shoot apices and anthers were collected at the same developmental stage as those for RNA-Seq. Values are the mean ± SD of three biological replicates.

**Supplementary** **Table S1.** **Summary of the transcriptome data.**

| **Sample** | **Raw reads** | **Clean reads (%)** | **Mapped reads (%)** | **Uniquely mapped reads (%)** |
| --- | --- | --- | --- | --- |
| **WT-SA_rep1** | 60,472,974 | 59,875,024 (99.01%) | 56,535,134 (94.42%) | 55,530,088 (92.74%) |
| **WT-SA_rep2** | 59,033,866 | 58,526,520 (99.14%) | 55,066,308 (94.09%) | 54,100,853 (92.44%) |
| **WT-SA_rep3** | 48,274,788 | 47,771,960 (98.96%) | 44,509,238 (93.17%) | 43,725,885 (91.53%) |
| **R10-SA_ rep1** | 46,277,352 | 45,981,740 (99.36%) | 43,817,455 (95.29%) | 43,042,706 (93.61%) |
| **R10-SA_ rep2** | 51,735,460 | 51,409,584 (99.37%) | 49,113,991 (95.53%) | 48,315,846 (93.98%) |
| **R10-SA_ rep3** | 55,212,556 | 54,878,128 (99.39%) | 52,585,082 (95.82%) | 51,709,097 (94.23%) |
| **WT-An_rep1** | 53,238,276 | 52,799,186 (99.18%) | 49,268,156 (93.31%) | 48,111,308 (91.12%) |
| **WT-An_rep2** | 51,866,940 | 51,456,134 (99.21%) | 48,087,901 (93.45%) | 47,158,892 (91.65%) |
| **WT-An_rep3** | 60,972,438 | 60,445,300 (99.14%) | 55,384,456 (91.63%) | 54,279,077 (89.8%) |
| **R10-An_ rep1** | 57,788,168 | 57,357,292 (99.25%) | 53,871,861 (93.92%) | 52,820,506 (92.09%) |
| **R10-An_ rep2** | 53,254,058 | 52,867,106 (99.27%) | 50,199,944 (94.95%) | 49,162,050 (92.99%) |
| **R10-An_ rep3** | 64,947,118 | 64,431,272 (99.21%) | 61,197,039 (94.98%) | 60,018,837 (93.15%) |

SA, shoot apex; An, anther.

**Supplementary Table S4. List of primers used in this study.**

| **Primer** | **Sequence** |
| --- | --- |
| **Primers for gene cloning** | |
| *SlMYB33*-F | 5'-ATGAGTATGACAAGTGAAAGCG-3' |
| *SlMYB33*-R | 5'-TCAGTACCAGATGGATCTTACA-3' |
| **Primers for qRT-PCR** | |
| *SlMYB33*-F | 5'-TCAACAACGCCTCCAGCCAAG-3' |
| *SlMYB33*-R | 5'-GAGCAAGCAAAGAACCATCATCC-3' |
| *SlGAMYB1*-F | 5'-ACTCCTCTTCAGAGCCCACA-3' |
| *SlGAMYB1*-R | 5'-AATGAAGGAAGAGCCGAATG-3' |
| *AN*-F | 5'-TTGAAGGGAATGGTAAGGTT-3' |
| *AN*-R | 5'-AGTTGAATGACTGAAAGGGC-3' |
| *FA*-F | 5'-TGCTAAGGAACGAGGTGAAA-3' |
| *FA*-R | 5'-AAATGGCTAGTCGAGGATGT-3' |
| *S*-F | 5'-TCTATCATTCAACAAAGACCTC-3' |
| *S*-R | 5'-GTAAAACACATTAGCATCACCT-3' |
| *SPGB*-F | 5'-CAAGACTCAAAACAATGGAA-3' |
| *SPGB*-R | 5'-AAGAAATGAAGCTCAGGAAC-3' |
| *FPF1*-F | 5'-TGACAAGAAAGGTGTTGCCCAT-3' |
| *FPF1*-R | 5'-CGAACTTCAAAAACCGATGGAT-3' |
| *FCA*-F | 5'-CGGCAAGTCATCATGTCTCT-3' |
| *FCA*-R | 5'-CATCCCCCTTCTTCCAGTTA-3' |
| *Lin7*-F | 5'-GTTTGGGCTCATTCCGTTTC-3' |
| *Lin7*-R | 5'-ACTTGGGTTTGGTTGGCATC-3' |
| *SPS*-F | 5'-CATCAAGCCAGGAGCCGAAG-3' |
| *SPS*-R | 5'-TCCCCCCCACAAAAACAACC-3' |
| *SUS3*-F | 5'-TGTTACTCGGCTTCTCCCTG-3' |
| *SUS3*-R | 5'-TTCTTTCCCCACATCCTCAA-3' |
| *Solyc12g040700*-F | 5'-GTGAAAGAAGGCATCCAAAACA-3' |
| *Solyc12g040700*-R | 5'-GCACACACATACCTCAAGCAGA-3' |
| *TPS*-F | 5'-TCCATTATCTCCCACTTCCGC-3' |
| *TPS*-R | 5'-CTCCTTCAGGTGTGCCCTCAA-3' |
| *Solyc04g015200*-F | 5'-TACCACAGAGCAGGACCTCG-3' |
| *Solyc04g015200*-R | 5'-TCCCAAAAATCTCACGAACG-3' |
| *Solyc07g065900*-F | 5'-GCACTCAATGACCAGCATGTTC-3' |
| *Solyc07g065900*-R | 5'-ATTTTCTTTTTTTCCACCCCAA-3' |
| *GLU*-F | 5'-TTGTTGAGGTTTTGTTCGTTTTA-3' |
| *GLU*-R | 5'-GTCAGCAATTTTTTCTGGATGTT-3' |
| *AGPase*-F | 5'-CTCCAATCTACACCCAACCTCG-3' |
| *AGPase*-R | 5'-AGTATCCGTCTGACTCCCTCGC-3' |
| *Solyc09g091030*-F | 5'-TGTGGAGAACACCGATGACG-3' |
| *Solyc09g091030*-R | 5'-ACCAATCCCCACCACACATC-3' |
| *SlSTP13*-F | 5'-CTATGGGCTTCAAGGGCAGT-3' |
| *SlSTP13*-R | 5'-GTTCAACCCTCCTGTATCCG-3' |
| *Solyc06g009190*-F | 5'-TATGCTCGTAAACCTAACCCG-3' |
| *Solyc06g009190*-R | 5'-TCAATGCAAAATCACCACTCC-3' |
| *Solyc05g047590*-F | 5'-AGAAGTGTTGTTGATGGTTGGA-3' |
| *Solyc05g047590*-R | 5'-AATGAGTGGGCGTAGAGAGTGT-3' |
| *PL*-F | 5'-CATCGCATTACGGGTGGAGA-3' |
| *PL*-R | 5'-TCAGGGGCAAGAAATCGGTT-3' |
| *PG*-F | 5'-GGCAGGGAGGTAGAGGGTATG-3' |
| *PG*-R | 5'-AGGTGGATTGTTTAGCCCTGAG-3' |
| *EF-1α*-F | 5'-GACAGGCGTTCAGGTAAGG-3' |
| *EF-1α*-R | 5'-CCAATGGAGGGTATTCAGC-3' |
| *miRNA159a*-F | 5'-CAGTGTCCAGAATCGGCTTAT-3' |
| *miRNA159b*-F | 5'-CAGGTAAATGTGAGGAGCAGAT-3' |
| *U6*-F | 5'-ACTGTTCATGCCATCACTGC-3' |
| **Primers for *in situ* probes** | |
| *SlMYB33*-Sp6-F | 5'-GATTTAGGTGACACTATAGAATGCTCACTCCAAAGTACGATTGCAAGTT-3' |
| *SlMYB33*-T7-R | 5'-TGTAATACGACTCACTATAGGGCAGATGGATCTTACAGTGTTTTGCA -3' |
| **Primers for RNAi construct** | |
| *SlMYB33*-I1-F | 5'-GGACTAGTCCAAAGTTGCCGTTTGCGATGGGCCAA-3' |
| *SlMYB33*-I1-R | 5'-CGGGATCCCGGGAGGCGTTGTTGAGAGGAATGACC-3' |
| *SlMYB33*-I2-F | 5'-AGGCGCGCCTACGCTGTTGGTGGTGGTAC-3' |
| *SlMYB33*-I2-R | 5'-CGATTTAAATGAGAGGGGTAAACAGTGAATC-3' |
| **Primers for identification of transformants** | |
| *35S*-F | 5'-CTCCTCGGATTCCATTGCCC-3' |
| *35S*-R | 5'-ATAGAGGAAGGGTCTTGCGAA-3' |
